# Surface Reconstruction of Fluoropolymers in Liquid Media

**DOI:** 10.1021/acs.langmuir.2c00198

**Published:** 2022-04-08

**Authors:** Eleanor Milnes-Smith, Corinne A. Stone, Colin R. Willis, Susan Perkin

**Affiliations:** † Department of Chemistry, Physical and Theoretical Chemistry Laboratory, University of Oxford, Oxford OX1 3QZ, United Kingdom; ‡ Defence Science and Technology Laboratory, Porton Down, Salisbury, Wiltshire SP4 0JQ, United Kingdom

## Abstract

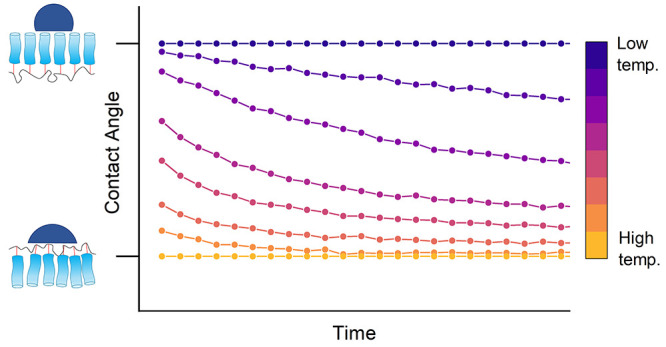

Surface reconstruction is the rearrangement of atoms or molecules at an interface in response to a stimulus, driven by lowering the overall free energy of the system. Perfluoroalkyl acrylate polymers with short side chains undergo reconstruction at room temperature when exposed to water. Here, we use contact angle aging to examine the liquid- and temperature- dependency of surface reconstruction of plasma polymerized perfluoroalkyl acrylates. We use a first order kinetic model to examine the dynamics of reconstructive processes. Our results show that, above the bulk melting point of the polymers, the contact angles of both polar and nonpolar (hydrocarbon) liquids show a time dependency well fit by the model. We conclude that surface reconstruction can be driven by the preferential segregation of hydrocarbon and fluorocarbon moieties as well as by polar interactions. This has implications in terms of using fluorocarbons to design oleophobic surfaces (and vice versa) and in terms of designing fluorocarbon and/or hydrocarbon surfaces with switchable wettability.

## Introduction

Reconstruction of polymer surfaces is a well-known phenomenon which has been of great interest for some time in developing so-called “smart” surfaces, which undergo changes in response to an applied stimulus, such as temperature, electric potential, humidity, or certain liquids.^1−9^ These are useful in a wide variety of contexts, including oil/water separation,^10−12^ anti-biofouling,^13,14^ and biomedical applications including drug delivery and implants.^15−18^ There are, however, also circumstances in which surface reconstruction results in a loss of desirable functionality.

**Figure 1:**
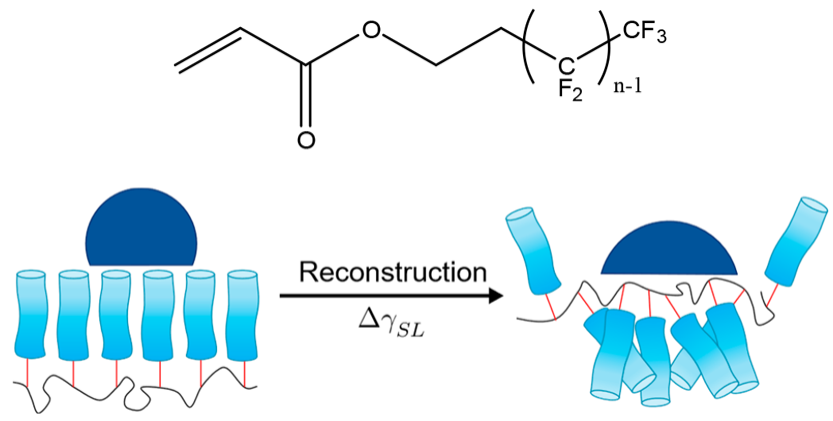
Chemical structure of the perfluoroalkyl acrylate (PFAC-*n*) monomer and Schematic showing reconstruction of a PFAC-*n* surface upon exposure to water. The blue cylinders represent the perfluoroalkyl chain, and the red sections represent the acrylate linkage to the backbone.

This paper focuses on perfluoroalkyl acrylate (PFAC-*n*, where *n* is the length of the perfluoroalkyl chain) polymers, which are used in industry to manufacture low surface energy materials. The generic PFAC-*n* monomer structure is shown in Figure 1. Coulson et al. developed a method using pulsed plasma polymerization to graft the PFAC-8 polymer to a variety of substrates.^19^ Plasma polymerization is favored by industry as it is a solvent-free process which can synthesize a polymer and graft it to a substrate in a single step.^20^ Because it is a gas phase process, it is independent of substrate geometry and produces uniform coatings with tunable thickness.^21^ Using the pulsed technique, rather than a more conventional, continuous wave plasma process has the additional advantage that fragmentation of the monomer species is kept to a minimum and the chemical structure of the polymer closely resembles those synthesized in the solution-phase.^22,23^ The coating developed by Coulson et al. is super-omniphobic and has been used successfully at the industrial scale to produce liquid repellent coatings for clothing, electronics, and filtration devices.^24,25^ However, compounds with fluoroalkyl chains of eight or more carbons are now known to bioaccumulate, meaning that shorter chain alternatives must be sought.^26,27^ The liquid-repellency of the PFAC-8 polymer hinges on the fact that the low surface CF_3_ groups preferentially segregate at the surface and the fluoroalkyl side chains crystallize, effectively locking the structure in place.^28,29^ Compounds with fewer than eight perfluorinated carbons are amorphous under ambient conditions. Work by Honda and co-workers on PFAC-*n* polymers and similar compounds has shown that these shorter chain (*n* < 8) analogues are prone to surface reconstruction when exposed to water.^30−32^ Strong polar interactions induce the migration of the acrylic groups to the surface and the liquid spreads. A schematic of this process is shown in the bottom half of Figure 1.

A number of other authors have used changes in contact angle and contact angle hystereses to examine the reconstruction of polymer surfaces,^30−35^ and a recent paper by Butt et al. proposed a simple first order kinetic model to relate changes in contact angle (θ) to spontaneous adaptation of a surface in contact with a liquid or vapor.^36^ Adaptation in this context could mean polymer reconstruction but may also refer to swelling of the polymer,^37−40^ adsorption of vapor/liquid on the surface (or replacement of adsorbed species),^32,41,42^ surfactants binding at both the solid–liquid and liquid–vapor interfaces,^43,44^ and ordering of the liquid molecules at the solid–liquid/liquid–vapor interfaces.^45,46^ These effects may be concurrent, and it may not be possible to separate out these effects. The time scale of adaptive processes is characteristic of the system, and measurements of surface properties such as wetting will necessarily mirror this time scale. The model proposed by Butt et al.^36^ assumes that the solid–liquid surface energy, γ_SL_, relaxes from its initial state, γ_SL_(0), to its final state, γ_SL_(∞), following first order kinetics such that
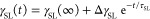
1where *t* is time, τ_SL_ is the characteristic relaxation time of the system, and
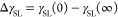
2The corresponding change in θ therefore varies with *t* such that
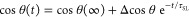
3where
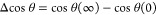
4

In addition to Butt’s first order model, the solid surface energies will also be evaluated using a more commonly used approach: the Owens–Wendt–Rabel–Kaelble (OWRK) method.^47,48^ This allows γ_SV_ to be calculated (subject to several assumptions) in the absence of liquid- and/or time-dependent effects. Here, it is assumed that surface energy can be split into two distinct contributions from polar and dispersive forces such that
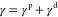
5It is further assumed that the solid–liquid surface energy, γ_SL_, is the geometric mean of the solid–vapor surface energy, γ_SV_, and the liquid–vapor surface energy, γ_LV_, such that
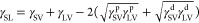
6

This equation may be combined with the Young equation and rearranged to give the linear equation
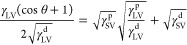
7allowing the values of γ_SV_^p^ and γ_SV_^p^ to be extracted by measuring the values of θ for two or more liquids with known values of γ_LV_^p^ and γ_LV_^d^.^49^ This is useful in the context of examining surface reconstruction as it is expected that the migration of the acrylate groups to the surface would result in an increase in γ_SV_^p^.

While the work by Honda et al. focused on the role of polar liquids at room temperature, the work in this investigation also encompasses the role of temperature in governing molecular mobility (and hence surface reconstruction), and the role of nonpolar liquids. Furthermore, whereas previous studies have focused on polymers synthesized in the solution phase, the work here focuses on polymers synthesized via pulsed plasma polymerization–which can be rather different in terms of morphology. Although this investigation is focused on a specific set of substrates, the findings can be generalized and applied to a wide range of systems.

In summary, this paper investigates the time-, temperature-, and liquid-dependency of contact angles on plasma polymerized perfluoroalkyl acrylates with variable side chain lengths. The paper is laid out in three parts: First, since wetting behavior is dependent on the chemical composition, topography, and phase behavior of a surface, a detailed characterization of the PFAC-*n* polymers has been performed using angle-resolved X-ray photoelectron spectroscopy (ARXPS), atomic force microscopy (AFM), and differential scanning calorimetry (DSC), as well as some basic contact angle measurements. Second, the time dependent contact angles, θ(*t*), at variable temperatures, *T*, are presented, and the questions of whether surface reconstruction has taken place and what drives surface reconstruction are addressed. Third and finally, the contact angle data is then examined using Butt’s first order model and is related to the kinetics of surface reconstruction.

## Materials and Methods

### Materials

The general structure for perfluoroalkyl-*n*-acrylate (PFAC-*n*) monomers and polymers is shown in Figure 1. Monomers with *n* = 6, 8, and 10 were obtained from Sigma-Aldrich Ltd. (≥97%), while the PFAC-4 monomer was obtained from Fluorochem Ltd. (≥97%). All monomers were purified prior to use by multiple freeze–thaw cycles.

### Surface Synthesis

Polymer films were prepared via pulsed plasma polymerization. An inductively coupled glass glow discharge reactor (10 cm diameter, base pressure < 1 × 10^–2^ mbar) was connected to an Edwards two-stage rotary vacuum pump via a thermocouple pressure gauge and a liquid nitrogen cold trap. Power was supplied from a 13.56 MHz radio frequency generator which was connected to a pulse generator, and to and L-C matching unit. The chamber and monomer inlet were also enclosed in an oven. Prior to use, the chamber was scrubbed with IPA and a 50 W air (2 × 10^–1^ mbar) plasma was run for 1 h.

The samples to be coated (glass slides for AFM, XPS, and contact angle measurements, ground glass for DSC) were placed in the chamber inside the coils and the whole system was evacuated to base pressure. Monomer vapor was then introduced at pressure of 2 × 10^–1^ mbar (room temperature for *n* = 4 and 6, 30 °C for *n* = 8, and 50 °C for *n* = 10). A brief, 30 s, continuous wave plasma was ignited (40 W) before the pulse generator (t_on_ = 40 μs, t_off_ = 20 ms) was switched on and the plasma run for a further 10 min (glass slides) or 2 h (ground glass). After this time, the RF generator was switched off and the monomer vapor allowed to flow for 5 min before the system was evacuated back to base pressure.

### Solid-State ^19^F NMR

Experiments were carried out using a Bruker Avance III spectrometer, operating at ∼376 MHz fF detection and using a 3.2 mm HFX probe. CFCl3 was used as an external reference. Spectra were acquired with 16 scans, having a 90° pulse width of 2.5 μs and a delay time of less than 10 s. All spectra were acquired using magic angle spinning (MAS) at a rate of 20 kHz.

### X-ray Photoelectron Spectroscopy (XPS)

Survey spectra were acquired using a Thermo Scientific K-Alpha spectrometer with a takeoff angle of 90° to the surface normal. Angle-resolved XPS (ARXPS) carbon 1s spectra were obtained using a Thermo Scientific Theta Probe; spectra were acquired in parallel at angles from 27.5° to 77.5° to the normal in 5° steps. To prevent damage to the surfaces, the X-rays were rastered across the surface in a 16 × 16 grid with 500 μm between points. At each point, the C 1s spectrum was measured for 60 s, giving a total acquisition time of 256 min. The spectra at all points were summed to give a single, angle-resolved spectrum. Data was analyzed using CasaXPS software.

### Differential Scanning Calorimetry (DSC)

A PerkinElmer PYRIS Diamond DSC was used in the thermal analysis of PFAC-*n* samples. The samples were loaded at −50 °C and equilibrated for 2 min. The samples were then heated from −50 to 150 °C at a rate of 10 °C min^–1^, held at 150 °C for 5 min, and then cooled to −50 °C at a rate of 10 °C min^–1^. The samples were held at −50 °C for 5 min, and the heating and cooling cycle was then repeated once more. Data was analyzed using the PYRIS software

### Atomic Force Microscopy (AFM)

Measurements were carried out using a Digital Instruments Multimode NanoScope IIIb in tapping mode. Tapping mode involves rastering an oscillating cantilever across the surface; the oscillations are then damped by interactions with the surface. The cantilevers used had a force constant of 40 N m^–1^ and a resonant frequency of 325 kHz. Data analysis was carried out in the NanoScope analysis program.

### Contact Angle (CA) Measurements

Initial room temperature contact angle measurements were performed using a Krüss DSA25 goniometer using water and *n*-dodecane as probe liquids. Static contact angles were measured by dispensing a 5 μL drop on the surface, while advancing and receding contact angles were measured by dispensing and then retracting a 5 μL drop at a rate of 0.5 μL s^–1^; the largest angle during dispensation and the smallest angle during retraction were taken as the advancing and receding CAs, respectively. Each measurement was repeated five times on three different samples, each made during a different deposition.

Variable temperature static contact angle measurements were carried out using a Krüss DSA100 goniometer fitted with a TC40 temperature-controlled chamber and operated under a low flow of dry air. Measurements were performed at temperatures from 20 to 100 °C at intervals of 10 °C. Prior to each measurement, the surfaces were allowed to reach thermal equilibrium over a period of 10 min and a thermal camera was used to ascertain whether this was the case. The probe liquids from room temperature experiments were exchanged for the less volatile ethylene glycol (“EG”, Sigma-Aldrich Ltd., 99.8%) and *n*-hexadecane (“HD”, Sigma-Aldrich, Ltd., ≥99%), and the drop volume was decreased to 2 μL to promote more rapid thermal equilibrium with the surface. The initial measurements of θ demonstrated that the experimental scatter was greater across different surfaces than across the same surface, so measurements were performed on a single, representative surface and repeated three times.

## Results and Discussion

### Characterization of PFAC-*n* Surfaces

#### Chemical Composition

Solid-state ^19^F NMR spectra were recorded for each of the PFAC-*n* polymers (*n* = 4, 6, 8, 10) in order to interrogate the local F environments in the different polymers. The spectra for all PFAC-*n* polymers (Figure S1.2) revealed contributions from four different fluorine environments: CH_2_—CF_2_, CF_2_—CF_2_, CF_3_—CF_2_, and C 1s CF_2_—CF_3_. Notably, there were no contributions in the solid-state ^19^F NMR spectra from C(CF_3_)_3_, CF(CF_3_)_2_, or CF moieties, which indicates that no observable fragmentation or cross-linking of the monomer species had taken place. Similarly, deconvolution of the XPS spectra acquired at a takeoff angle normal to the surface, shown in Figure S1.2, reveal contributions from six carbon environments; CF_3_, CF_2_, C=O, C—O, C—CF_*n*_, and C—CH_*n*_. This is also consistent with no observable fragmentation of the monomer species having occurred during the deposition process and indicates that the pulsed plasma technique has produced polymers which are chemically similar to those produced via solution-phase synthesis.

**Figure 2:**
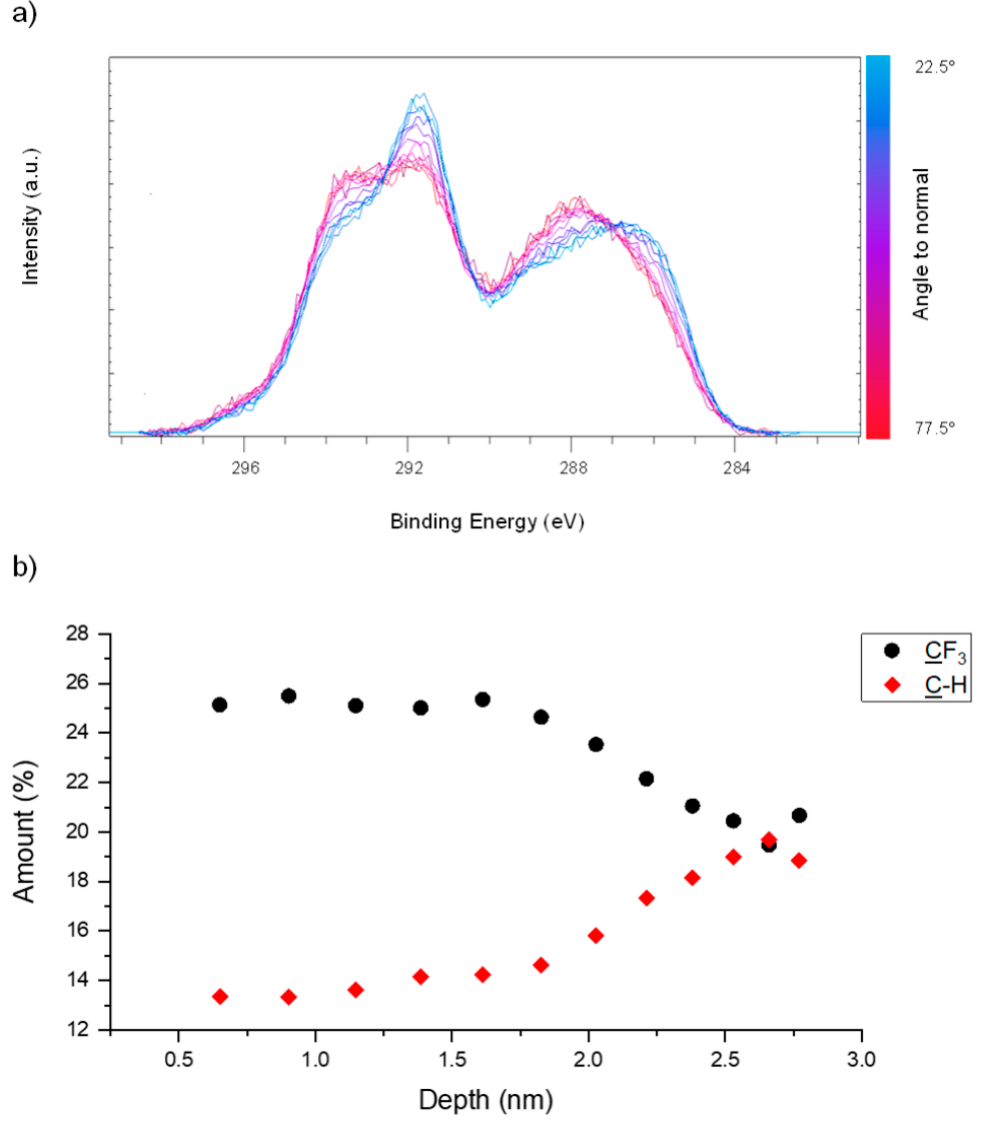
(a) Angle-resolved C 1s XPS spectra of a PFAC-6 polymer surface acquired in parallel at angles between 22.5° (blue) and 77.5° (red) to the surface normal at intervals of 5°. To account for the decrease in signal intensity, a Shirley background subtraction was performed, and the area under the peaks was normalized. (b) Variation with penetration depth, *d*, of the concentration of the CF_3_ and C—CH_*n*_ species. Penetration depth is defined as the depth at which 63% of the signal originates and is estimated as *d* ∼ λ sin θ where θ is the takeoff angle relative to the surface and λ is the electron inelastic mean free path. In fluoropolymers where the photoelectrons are excited from the C 1s level by Al Kα radiation, λ ∼ 3 nm.^50^

Angle-resolved XPS carbon 1s spectra were carried out for each of the PFAC-*n* polymers (*n* = 4, 6, 8, 10), in order to interrogate the local chemical composition of the surface and the variation of composition with depth. An example spectrum for PFAC-6 is shown in Figure 2 along with a plot of the variation in concentration of the CF_3_ and C—CH_*n*_ species, which sit at opposite ends of the monomer unit–the full data can be viewed in Figures S1.3 and S1.4.

All the PFAC-*n* polymers show a variation in composition within the 2 nm from the interface toward the bulk. The concentration of CF_3_ is approximately anticorrelated to that ofCH_n_ and is found in excess at the surface. The anticorrelation of the concentration of the functional groups at opposite ends of the monomer unit suggests the side chains have a general orientation with the CF_3_ groups toward the surface, and the other end of the side chain toward the bulk. However, the concentrations of the other C 1s environments (shown in the Supporting Information) display a far more complex behavior that would be expected in the case that the side chains are completely straight and/or aligned perfectly perpendicular to the surface. Rather, they likely possess some degree of tilt and flexibility, and variation in orientation. Unfortunately, accounting for this disorder significantly complicates any quantitative analysis of these results and for this reason we have opted for a solely qualitative discussion.

PFAC-4 overall displays less depth-dependent behavior than the other three polymer surfaces. Having fewer carbon units in the side chain reduces the strength of the intermolecular interactions between side chains and reduces the enthalpic benefit of side chain crystallization.

#### Thermal Characterization

**Figure 3:**
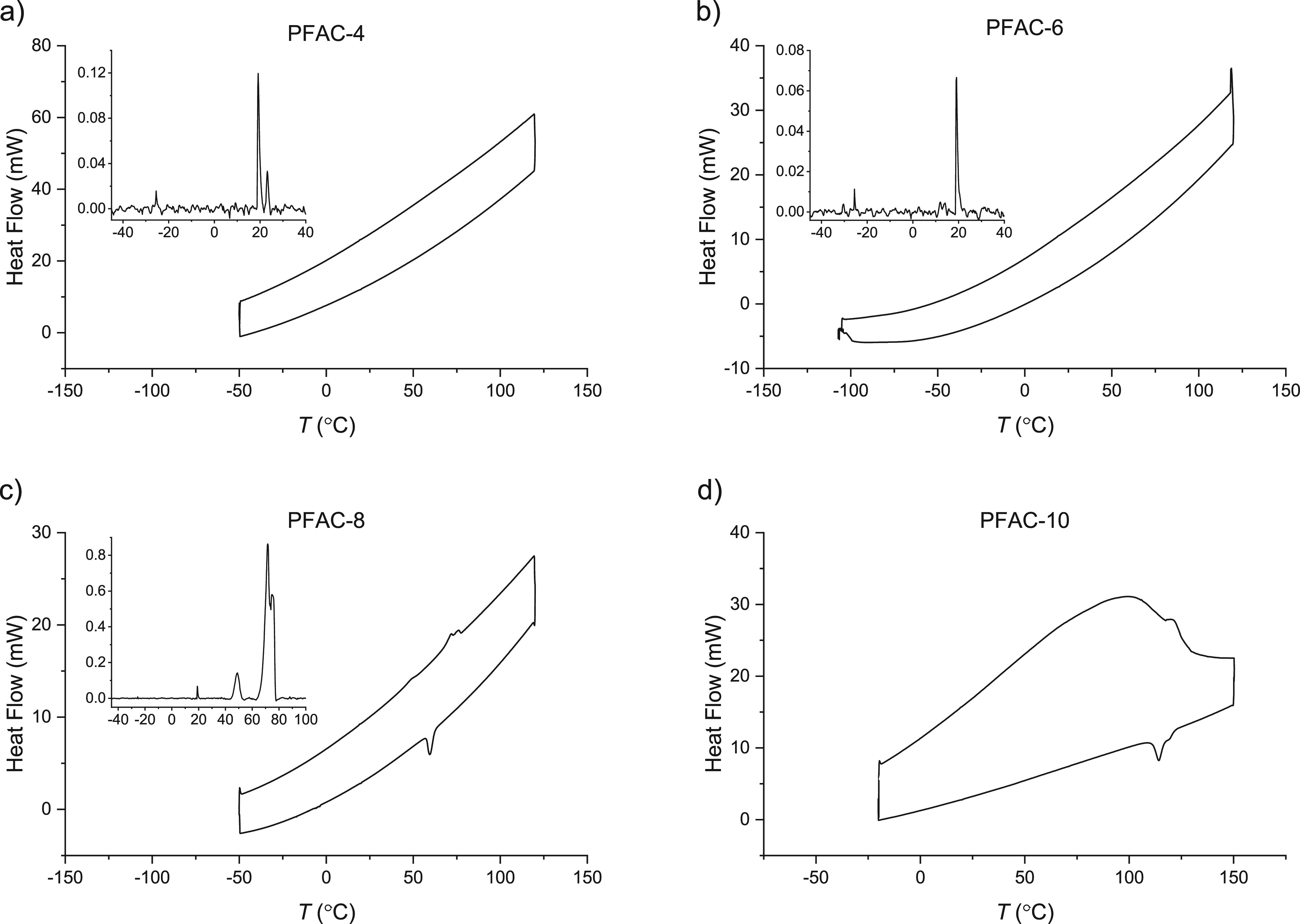
DSC traces (endothermic up) for the first heating and cooling cycle of (a) PFAC-4, (b) PFAC-6, (c) PFAC-8, and (d) PFAC-10 polymers. Background subtracted spectra are included as an inset. The difference between the heating and cooling curves gives an indication of which transitions are reversible. In the case of PFAC-4 and -6, all peaks that appear in the heating cycle are found in the cooling cycle, while PFAC-8 and −10 show significant differences between the two, indicating that they are kinetically trapped in nonequilibrium states.

In order to interrogate the phase state and phase transitions of the PFAC-n polymers we performed DSC measurements. Figure 3 shows the first heating (top curve) and first cooling (bottom curve) cycle of the PFAC-n polymers; background subtracted spectra are shown inset for PFAC-4, 6, and 8 to allow the lower intensity peaks to be clearly shown. The first heating cycle corresponds to the behavior investigated during the variable temperature contact angle aging experiments, while the cooling curve shows which of these transitions are reversible, thermodynamic transitions–second heating curves can be viewed in the Supporting Information.

The first heating cycle of PFAC-10 shows a broad melting peak across a wide range of temperatures–from below 0 °C up until its crystalline melting point which disappeared after the first cycle and resolved into a double peak at 123 and 126 °C and minor peaks at −25 and 19 °C (too small to be visible by eye in this graph but shown in the Supporting Information). PFAC-8 also displays irreversible melting transitions, with multiple, broad peaks in the first heating cycle which resolve into a single, major peak at 70 °C and minor peaks at 19 and −25 °C after the first heating cycle. This indicates that, having been deposited below their crystalline melting points, PFAC-8 and -10 are initially kinetically trapped in a disordered solid state at room temperature. After the first heating cycle, the equilibrium, reversible phase behavior emerges. In contrast, PFAC-4 and -6 displayed only reversible melting transitions in their phase behavior with melting peaks at −19 and 20 °C for both PFAC-6 and -4 and an additional melting peak at 19 °C for PFAC-4. The commonality of the low intensity peaks at −25 and 20 °C across all four PFAC-*n* polymers suggests that these transitions are linked to the polymeric backbone rather than the side chains. The additional peaks shown at 123 and 126 °C for PFAC-10 and 70 °C for PFAC-8 correspond to literature values for the melting of the crystallized fluoroalkyl side chain.^25^

#### Topography

The roughness and topography of the polymer surfaces in air was studied using AFM imaging. Micrographs of PFAC-8 and −10 polymer surfaces are shown in Figure S1.7. The root-mean-square (RMS) roughness values of these micrographs (each 20 × 20 μm^2^) are 38 nm (PFAC-8) and 34 nm (PFAC-10). The phase data for both of these surfaces indicated full coverage of the underlying glass substrate. Having undergone a melt below room temperature (Figure 3), the softness and smoothness (within the noise level of the instrument) of the PFAC-4 and -6 surfaces made it impossible to acquire clear images, even when using a cantilever with an extremely low spring constant.

#### Wetting Behavior (Room Temperature)

**Figure 4:**
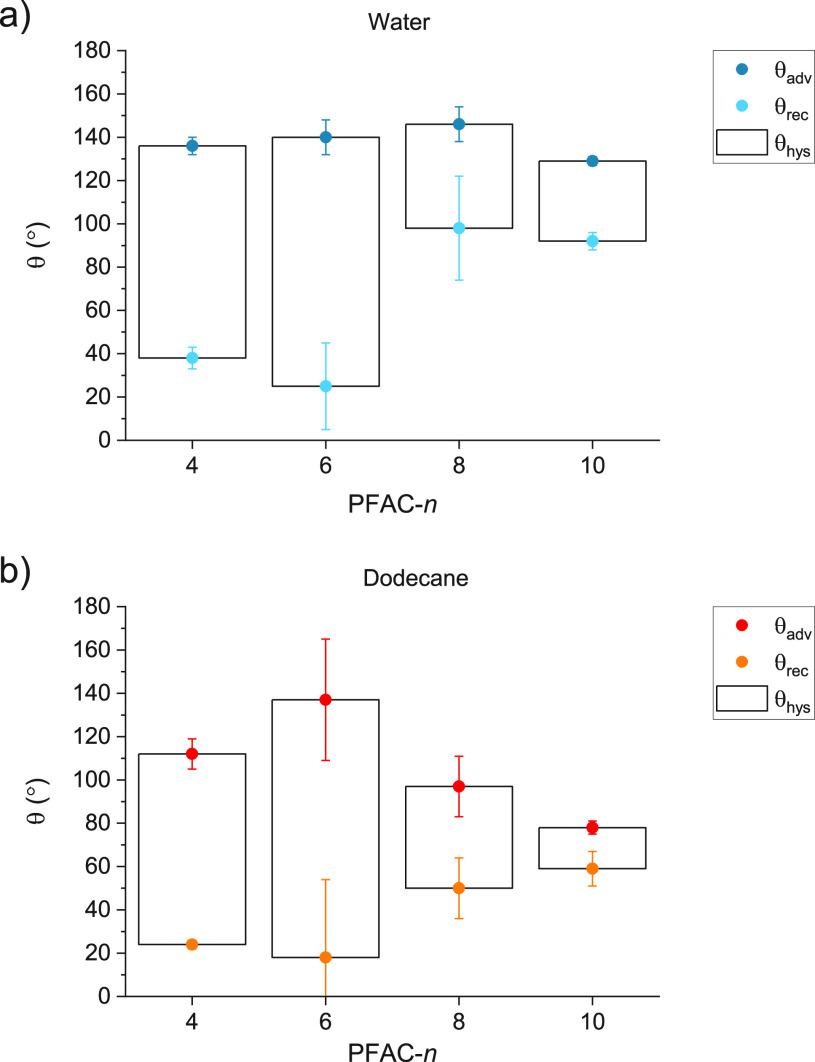
Advancing (dark) and receding (light) contact angles of (a) water and (b) dodecane on PFAC-n surfaces. CA hysteresis (bar) is the difference between these two values. Error bars represent one standard deviation from the mean

We next investigated the advancing and receding contact angles (θ_adv_ and θ_rec_, respectively) of water and dodecane on the PFAC-*n* polymer coatings. Figure 4 shows these values along with the difference, or hysteresis (θ_hys_), between them of water at room temperature (between 20 and 22 °C). The error bars, which represent one standard deviation from the mean across 15 measurements (5 measurements on 3 surfaces), are dominated by the differences between individual surfaces, rather than the variation between the measurements on a single surface. The values of θ_adv_ for water (top panel) are similar across the series of PFAC-*n* polymers. This implies that a maximum value of θ_adv_ has been reached, which is consistent with the Wenzel wetting regime, which gives a maximum contact angle of ∼150°.^51,52^ The behavior of dodecane, which has lower surface tension, is therefore more revealing of surface energy trends. For dodecane (bottom panel), the values of θ_adv_ show clear variation across the series with the maximum at *n* = 6 and decreasing values of θ_adv_ for shorter and longer chains in the order *n* = 6 > 4 > 8 > 10. This trend runs contrary to the literature, where γ_*SV*_ decreases with increasing *n*.^30^ We emphasize at this point that the large values of θ_hys_ for the PFAC-*n* surfaces, and the roughness of the PFAC-8 and -10 surfaces as measured via AFM indicate that the surfaces are not ideal (smooth and chemically homogeneous) with respect to wetting. Any discussion of surface energy (both γ_SV_ and γ_SL_) in this context is therefore a discussion of the *effective* surface energy: the combined effect of the intrinsic surface energy of the substance and the effect of roughness or heterogeneity. Similarly, it may no longer be the case that the measured, static contact angle is equivalent to the equilibrium contact angle defined by the Young equation. Henceforth, θ will be used to describe the measured, static contact angle and the comparison of this value with the equilibrium contact angle will be discussed on a case-by-case basis. The presence of CA hysteresis indicates that a surface is heterogeneous but does not indicate whether that heterogeneity is physical/topographical or chemical in nature. However, it is striking that PFAC-4 and -6, which are liquids at room temperature and are therefore smooth, have larger CA hystereses than PFAC-8 and -10, which are rough. This suggests a molecular-level or chemical heterogeneity of these surfaces, as may arise from surface reconstruction induced by the presence of the liquid droplet on the surface. In this case, surface reconstruction would mean that the triple-phase line advances across a low energy fluorocarbon surface but recedes across a higher energy acrylic surface–resulting in a large contact angle hysteresis.

### Contact Angle Aging

**Figure 5:**
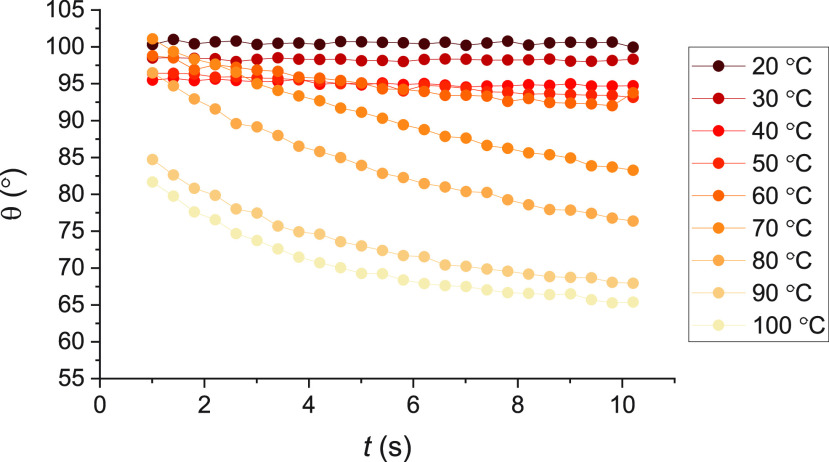
Variation of the static contact angles, θ, with time, *t*, of HD on PFAC-6. The data here is for a single representative drop at each temperature.

With this hypothesis in mind, we next investigated the possibility of molecular-level reconstruction of the polymer surfaces driven by interactions at the polymer-fluid interface. We measured the evolution of the contact angle, θ, of a sessile drop over time, *t*, for ethylene glycol (EG) and hexadecane (HD) on PFAC-*n* at temperatures 20 ≤ *T* ≤ 100 (as discussed in the Materials and Methods, it was necessary to exchange water and dodecane for less volatile liquids). An example plot showing the variation of the static contact angles, θ, with time, *t*, of HD on PFAC-6 at different values of *T* is shown in Figure 5; full data may be viewed in the Supporting Information.

**Figure 6:**
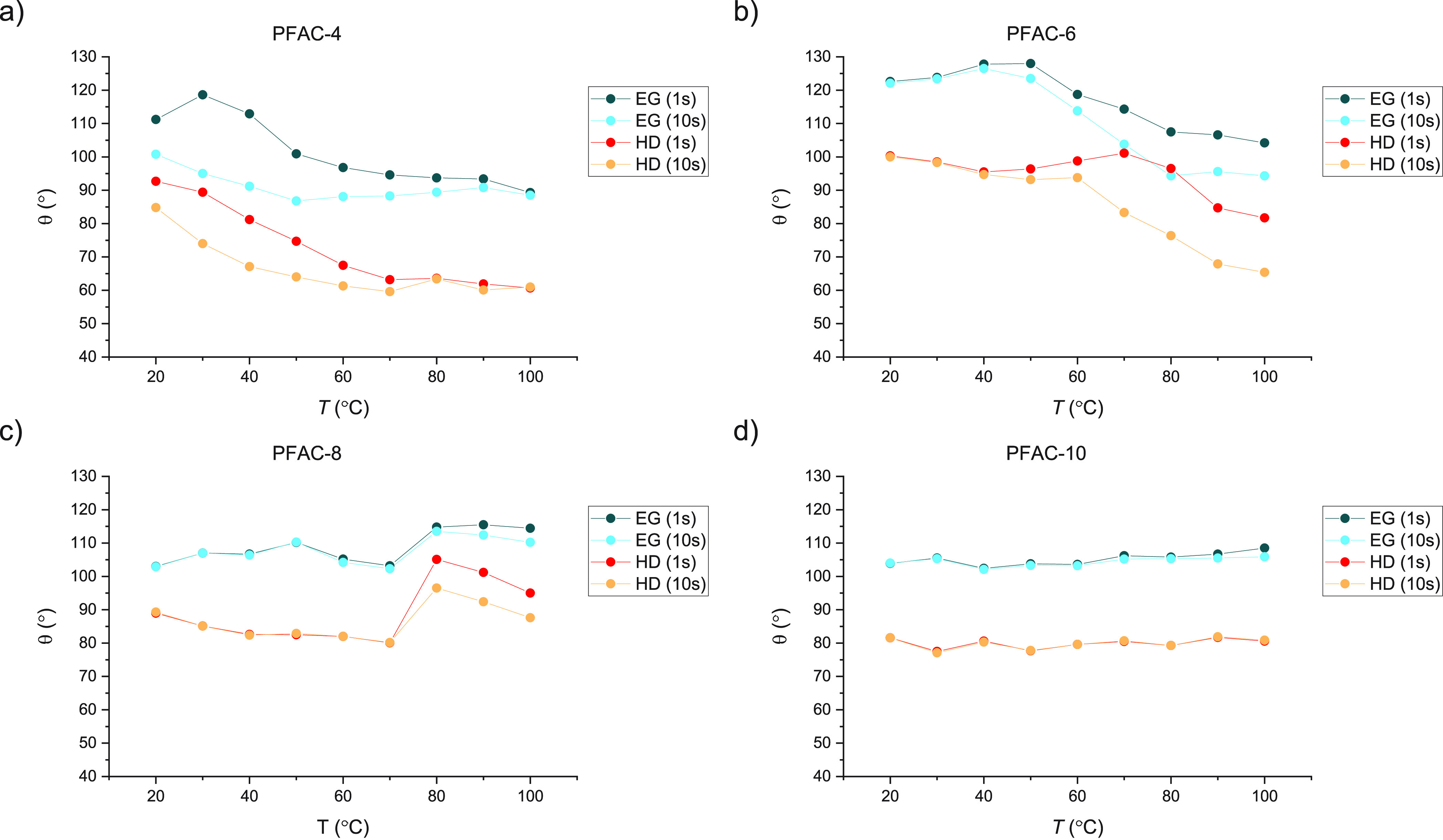
Values of θ of ethylene glycol (EG, blue) and hexadecane (HD, orange) at *t* = 1 (dark) and *t* = 10 (light) on (a) PFAC-4, (b) PFAC-6, (c) PFAC-8, and (d) PFAC-10polymer surfaces. The values are for a single representative drop at each temperature.

Figure 6 plots the values of θ for EG (blue) and HD (orange) at *t* = 1 (dark) and *t* = 10 (light), which allow the time-dependent behavior to be more clearly seen. The values shown are for a single, representative drop at each value of *T*; averages across all three measurements may be viewed in the Supporting Information. Overall, with the exception of PFAC-10, time-dependent wetting behavior was observed on all PFAC-*n* surfaces at some point across the temperature range in this experiment (20 ≤ *T* ≤ 100). However, both the onset and characteristics of this time-dependence vary by surface. Both EG (polar) and HD (nonpolar) exhibit this behavior, with similar onset temperatures for both liquids on a given surface Both PFAC-10 and −8 behave in the same way below their bulk, crystalline melting points with little or no dependence on *t*. Once PFAC-8 achieves the liquid state, the values of θ increase sharply and start to display a dependence on *t*. For PFAC-6, the values of θ also show no dependence on *t* in the low-*T* regime, a behavior which persists until *T* ≈ 40. At *T* > 40, a dependence on *t* emerges with the divergence between the values at *t* = 1 and *t* = 10 increasing with increasing *T*. PFAC-4 follows the opposite behavior to PFAC-6 in that the values of θ show a dependence on *t* in the low-*T* regime which then disappears as *T* increases. For both liquids on PFAC-4, the values of θ at *t* = 1 decrease with increasing *T*, before starting to plateau as the values of θ at *t* = 1 and *t* = 10 start to converge. An interesting observation is that, examining the figures for PFAC-6 and −4 side by-side, the wetting behavior in the high-*T* regime for PFAC-6 appears to map on to the low-*T* regime for PFAC-4. The following paragraphs will be devoted to providing an explanation for these phenomena.

**Figure 7:**
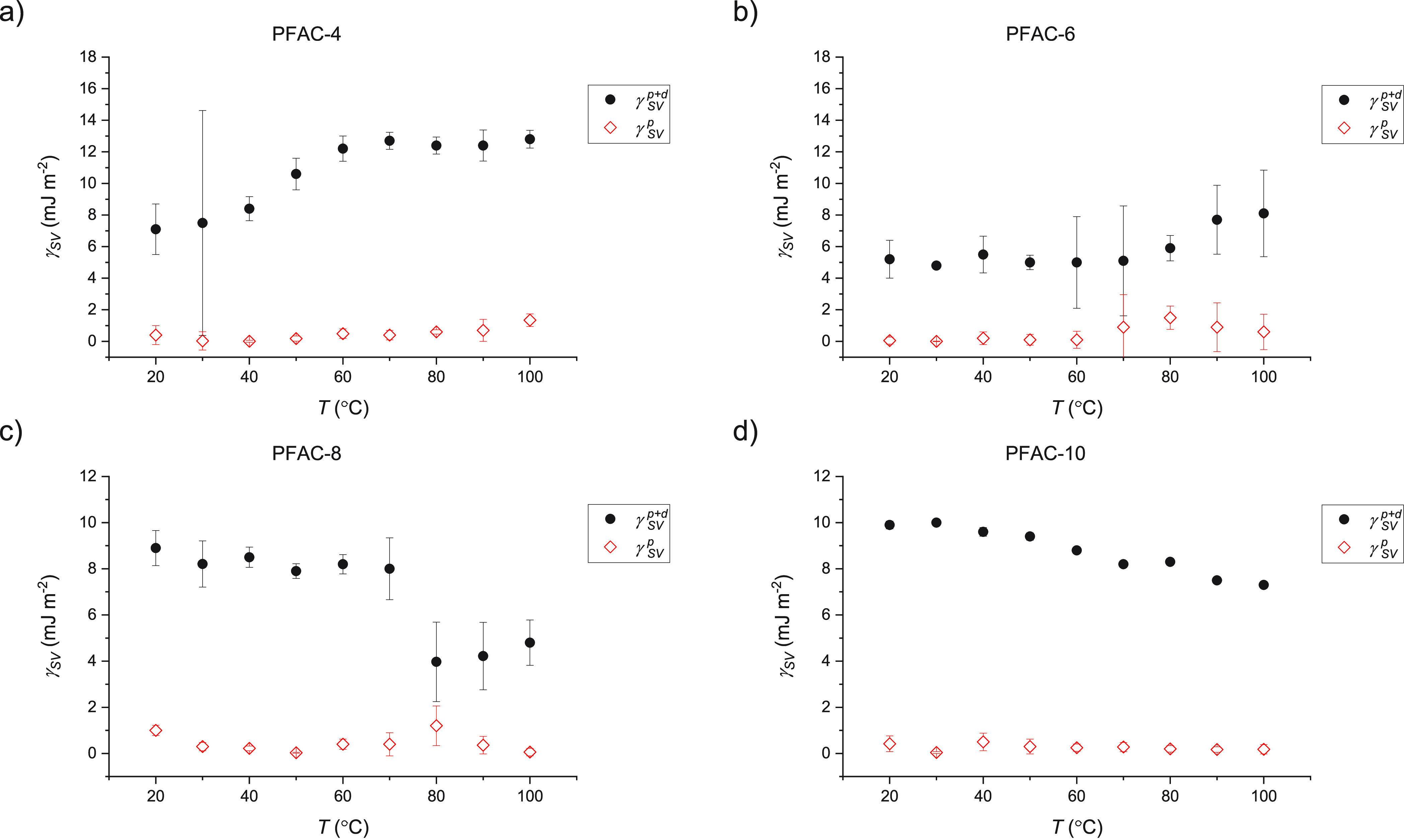
Total (black) and polar (red) surface energies of (a) PFAC-4, (b) PFAC-6, (c) PFAC-8, and (d) PFAC-10 from 20 to 100 °C. These values were estimated using the OWRK method as outlined in the introduction and assuming that the contact angles measured at 1 s are a reasonable substitution for the values at 0 s. The dispersive surface energy is the difference between the total and polar values.

We will first focus on the effect of temperature on the surfaces in the absence of liquid. We have estimated the values of γ_SV_ where 20 ≤ *T* ≤ 100 using the OWRK method outlined in the introduction. For now, we use the value of θ at *t* = 1 as the approximate value of θ at *t* = 0 (i.e., before surface rearrangement or other effects can take place), and the results are shown in Figure 7. This is a reasonable assumption where contact angle aging effects are slow or nonexistent but loses its validity in regimes where aging is rapid.

**Figure 8:**
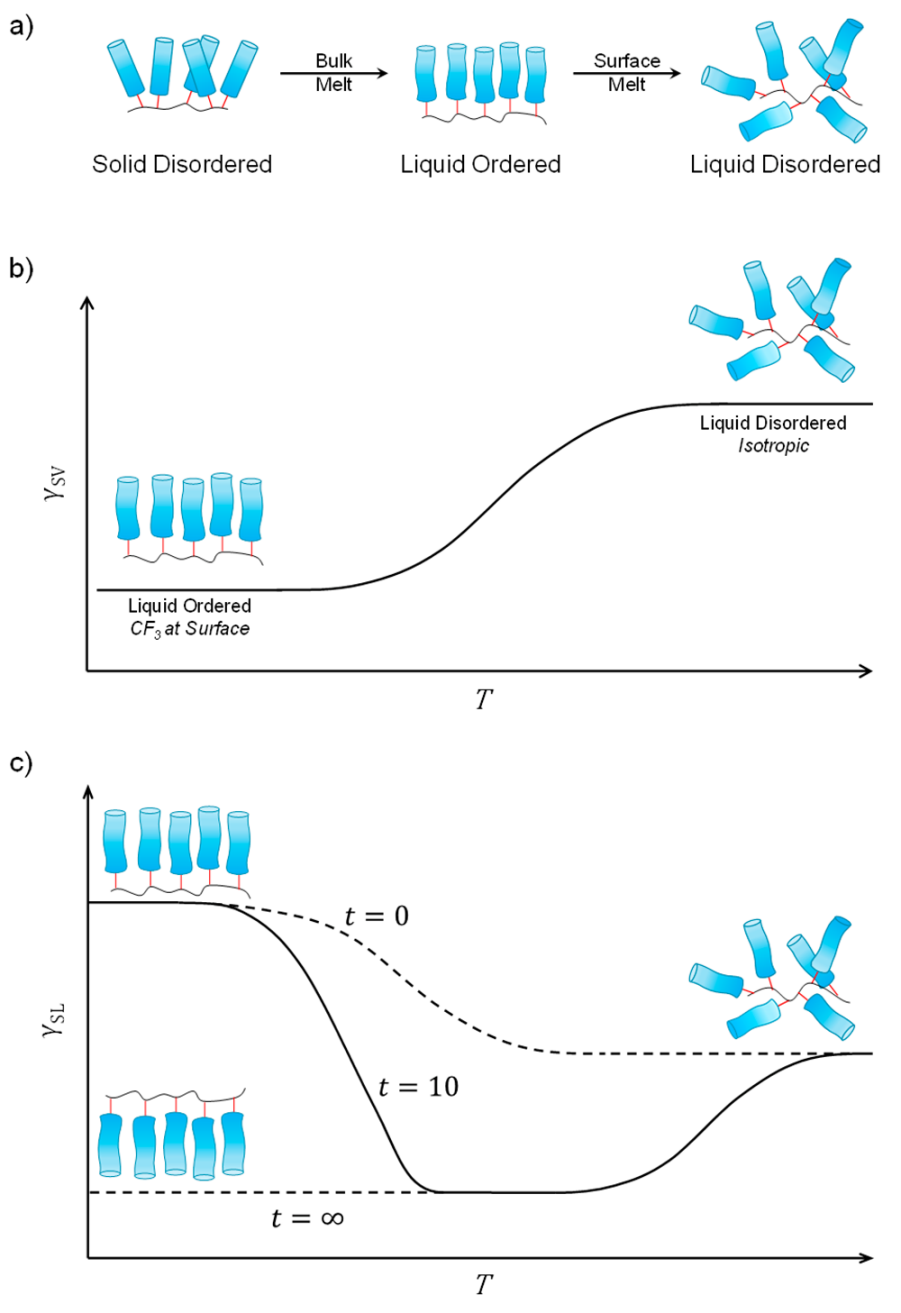
(a) Changes in side chain orientation at the PFAC-*n* surface across solid–liquid and liquid–liquid phase transitions. (b) Changes in γ_SV_ of PFAC-*n* across transition from liquid ordered (L_O_) to liquid disordered (L_D_) phase. (c) Summary of changes in γ_SL_ of PFAC-*n* with time and with temperature.

The totals values of γ_SV_ range between 6 and 13 mJ m^–2^ which match the range of values measured elsewhere of PFAC polymers produced via more conventional means.^53−55^ Below their crystalline melting points, the solid surfaces show a decrease in γ_SV_ with increasing *T*. In the absence of phase transitions, reconstruction, or other adaptations; the adhesion between the solid surface and probe liquid decreases as random thermal motion start to dominate. Although it is expected that solid surface energy will decrease somewhat with temperature, the decrease in liquid surface energy contributes more strongly to this trend.^56^ This serves as a useful reminder that the values calculated using the OWRK method are only ever an estimate of solid surface energy but are nonetheless useful for identifying trends and making comparisons between surfaces, even if the absolute values are subject to error PFAC-8 mirrors the properties of PFAC-10 up to its melting point (*T*_m_ = 72), at which point there is a discontinuity and transition to lower value of γ_SV_.This transition and its likely origin are interesting, and we map out a likely mechanism in the top and middle panels of Figure 8. The value of *T*_m_ as determined by DSC is the temperature at which the *bulk* achieves isotropy, but the segregation of the CF_3_ groups at the surface is sufficiently energetically favorable that the surface does not achieve full isotropy until higher temperatures.^28^ Genzer et al. examined a polymer with a C8 perfluorinated side chain and a bulk melting point of *T*_m_ = 70 and found that the surface had still not achieved isotropy even at 100 °C.^29^ Thus, we propose, the initial drop in γ_SV_ of PFAC-8 upon melting is because the molecules become sufficiently mobile to orient themselves to their lowest energy configuration (solid disordered, S_D_, to liquid ordered, L_O_, state). Then, as *T* increases further, γ_SV_ starts to increases again with temperature as entropic effects begin to dominate and the surface tends toward isotropy (liquid ordered, L_O_, to liquid disordered, L_D_, state); this is a more gradual transition compared to the sharp solid–liquid melt. A schematic of these three states are shown in Figure 8, along with the projected changes in γ_SV_ with temperature for the L_O_ to L_D_ transition. Having undergone a melt below room temperature, PFAC-4 and -6 already exist on the continuum between L_O_ and L_D_, with the longer-chain PFAC-6 tending toward a more ordered structure. The total values of γ_SV_ for PFAC-4 and −6 measured at *t* = 1, which are a reasonable approximation of the values in air, map on to the predicted curve, with PFAC-6 mapping onto the low-*T* regime and PFAC-4 mapping onto the high-*T* regime. However, it must be noted that the measured polar surface energies, γ_SV_^p^, change very little with *T*. At first glance, this would seem to lend itself to the conclusion that molecular reorientation cannot be responsible for the changes in γ_SV_ because the increased presence of polar acrylate groups at the surface should necessarily result in γ_SV_^p^ making up an increased proportion of γ_SV_. However, in this case, the choice of probe liquids obscures this effect–this will be discussed in the context of liquid-induced changes to the surface.

Having established evidence for the variation of PFAC-*n* contact angles and surface energies with time, temperature, and *n*, we now consider the likely underlying molecular interactions between the two probe liquids, EG and HD, and the PFAC-*n* surfaces which drive the effect. In general, the values of θ for both ethylene glycol (EG) and hexadecane (HD) on PFAC-*n* surfaces show a dependence on *t* and *T* on the whenever the polymer is above its melting point (*T* > *T*_m_). The liquid surfaces can be reasonably assumed to be smooth, and so topographical effects do not come into play. This is reinforced by the fact that PFAC-4 and -6 display the largest values of θ_hys_ at room temperature (*T* ≈ 20) and if this effect was due to pinning of the triple phase line on physical features, drop spreading would be inhibited. Contact angle aging effects are therefore solely a result of chemical changes, or molecular rearrangements, of the surface and it can be concluded that both polar (EG) and nonpolar (HD) liquids are able to drive surface reconstruction. In the case of EG and other polar liquids, this is already well reported in the literature and is the result of strong polar interactions between the liquid and the acrylate group.^57−60^ The acrylate group migrates to the surface, lowering the solid–liquid surface energy and resulting in the drop spreading on the surface. In the case of HD, we propose that the time dependency of the CAs is the result of the preferential segregation of the hydrocarbon and fluorocarbon moieties. Fluorocarbon-hydrocarbon interactions are particularly weak in comparison to hydrocarbon-hydrocarbon or fluorocarbon-fluorocarbon interactions.^61−63^ Segregation of the two moieties occurs in both liquid and solid systems of semifluorinated alkanes^64−66^ and in copolymer systems containing both fluorocarbon and hydrocarbon side chains^ ^67,60a^−68^ and it is perhaps a similar effect that drives the spreading of HD on PFAC-*n*. This would also account for the lack of change in the measured values of γ_SV_^p^ across the experimental temperature range (shown in Figure 8). To observe an increase in the measured value of γ_SV_^p^, there needs to be a larger increase in the interactions between the polar probe liquid and the surface than between the nonpolar probe liquid and the surface. Because both the dispersive and the polar probe liquids used in this study trigger surface reconstruction (albeit for different reasons), this effect is obscured.

The bottom panel of Figure 8 shows a model outlining how the reconstruction may vary with time and temperature for polymers above their melting temperature, consistent with the present measurements. Here, we show the change in side chain orientation with temperature at *t* = 0, *t* = 10, and *t* = ∞ and the consequent change in γ_SL_. At *t* = 0, the configuration of the solid–liquid interface is equivalent to that in air and so the shape of the curve is simply an inversion of the curve in the middle panel (as we are now examining solid–liquid surface energy, γ_SL_, rather than solid–vapor surface energy, γ_SV_). At *t* = ∞, the surface has undergone liquid-induced reconstruction and has relaxed into its lowest energy state. As with the configuration at *t* = 0, entropic effects dominate at high temperatures and the surface tends to isotropy. The stronger solid–liquid interactions in comparison to solid–vapor interactions mean that this transition is likely to occur at a higher temperature than in air and it is further likely that there is a degree of liquid dependency. For simplicity, we have assumed that the most enthalpically favorable configuration of the surface in both liquids is with complete inversion of the side chain. The line at *t* = 10 indicates the progression of the solid–liquid interface within the experimental time frame. In the low-*T* regime, the surface is kinetically trapped in its initial configuration and is unable to reconstruct; the line at *t* = 10 therefore maps onto the line at *t* = 0. In the context of our experimental results, this corresponds to the behavior of PFAC-6 at low temperatures. As *T* increases and we reach the mid-*T* region, molecular mobility increases, and the surface is able to reconstruct. Initially, the relaxation time is long, and so the surface undergoes only small changes during the experiment time. As *T* increases yet further, the relaxation time decreases, and the difference between *t* = 10 and *t* = 0 increases. This corresponds to the contact angle aging behavior of PFAC-6 at high temperatures and the behavior of the more mobile PFAC-4 at low temperatures. Eventually, the surface is able to attain its equilibrium state within the experimental time frame and the line at *t* = 10 maps onto the line at *t* = ∞. In the context of the experiment, this behavior corresponds to the behavior of PFAC-4 where the contact angles converge at high temperatures. Focusing again on the bottom panel of Figure 8 and the high-*T* region, the line for *t* = 10 continues to map onto the curve for *t* = ∞ as the surface tends toward isotropy at which point any time-dependent effects are eliminated.

Having concluded the surface reconstruction is responsible for the time-dependence of the contact angles of EG and HD and having discussed the concept of relaxation time in a qualitative sense, the next section will use Butt’s first order model to investigate the kinetics of surface reconstruction in a more quantitative fashion.

### Kinetics of Surface Reconstruction

Contact angle data was fitted to eq 3 and to achieve a good fit, the relaxation time, τ, needs to fall within the time frame of the experiment, such that 1 ≲ τ ≲ 10. Instances where the value of τ falls outside of these parameters (whether as a single relaxation process, of part of multiple relaxation processes) cannot be explored. For PFAC-4, the data could be fitted for temperatures 40 ≤ *T* ≤ 80 for EG, and 30 ≤ *T* ≤ 70 for HD. For PFAC-6, the data could be fitted for 80 ≤ *T* for EG, and 70 ≤ *T* for HD. None of the data for PFAC-8 or -10 could be fitted. In all cases, the successful fits had an adjusted *R*^2^ > 0.96 (mostly >0.98) and χ^2^ ≤ 6 × 10^–5^. The complete set of fitted data can be found in the Supporting Information, along with the values of *R*^2^ and χ^2^.

**Figure 9:**
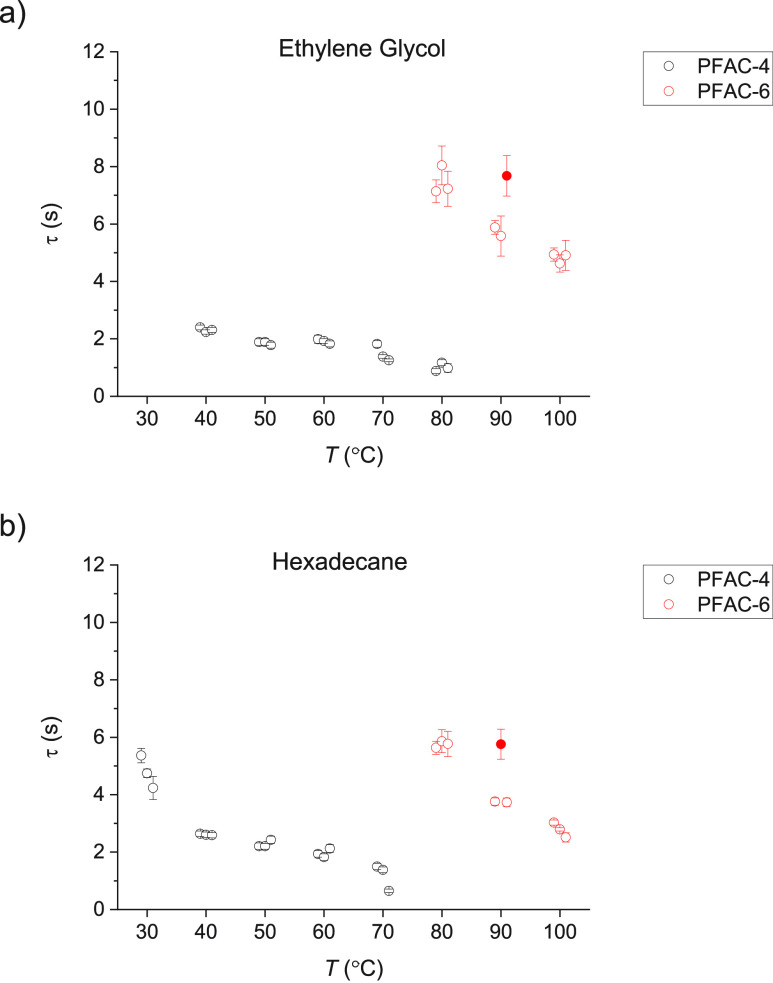
Relaxation times, τ, of (a) EG and (b) HD on PFAC-4 (black) and -6 (red). Error bars indicate goodness of fit, and where one result differs substantially from the other two, it is indicated by an unfilled circle.

Figure 9 shows the values of τ for EG and HD on PFAC-4 and −6 at different temperatures. The results for all three measurements carried out at each temperature are plotted separately, with the error bars representing the goodness of fit. In some cases (EG on PFAC-6 at *T* = 90, HD on PFAC-6 *T* = 90, HD on PFAC-4 at *T* = 70), one of the measured values is significantly different to the other two; where this occurs, the point is plotted as a filled circle. The appearance of these outliers suggests that the surfaces have some degree of inhomogeneity such as the presence of patches of adsorbed contaminants. As expected, there is a decrease in τ with increasing *T* across both liquids on both surfaces as molecular mobility increases. The curves for both liquids on PFAC-6 are shifted to higher temperatures than PFAC-4 and only overlap at two points; *T* = 80 for EG and *T* = 70 for HD. At both of these points, the value of τ for PFAC-4 is substantially shorter than for PFAC-6; the shift in the curve position suggests that this is the case across the temperature range studied and this is as expected for a shorter side chain. Values of τ for HD are shorter than EG at the same temperature on both surfaces. There is also a lower onset temperature for curve fitting, which also suggests that τ is shorter for HD than EG.

As discussed in the Introduction, there are various adaptive processes which can occur at the surface, such as reconstruction, polymer swelling, or liquid ordering, and the model does not differentiate between these processes. However, their characteristic time scales are very different; molecular liquid ordering occurs on a ns time scale, polymer swelling on a ms time scale, and polymer reconstruction can occur on anything between the s to day time scale.^68−70^ Overall, it seems that the relaxation times measured in this investigation are associated with reconstruction at the polymer surface. This process was not found to follow Arrhenius behavior.

In addition to determining the relaxation times of the PFAC-*n* surfaces, it was also possible to extrapolate the values of θ at *t* = 0 and *t* = ∞ (θ(0) and θ(∞), respectively). These values for EG and HD on PFAC-4 and -6 are shown in the Supporting Information. It is interesting to note that the calculated values of θ(∞) had a very small standard deviation in comparison with the measured values of θ(0) and despite the appearance of more than one characteristic value of τ at several temperatures. This indicates that despite some initial inhomogeneity in air, the surfaces tend toward homogeneity in liquid.

**Figure 10:**
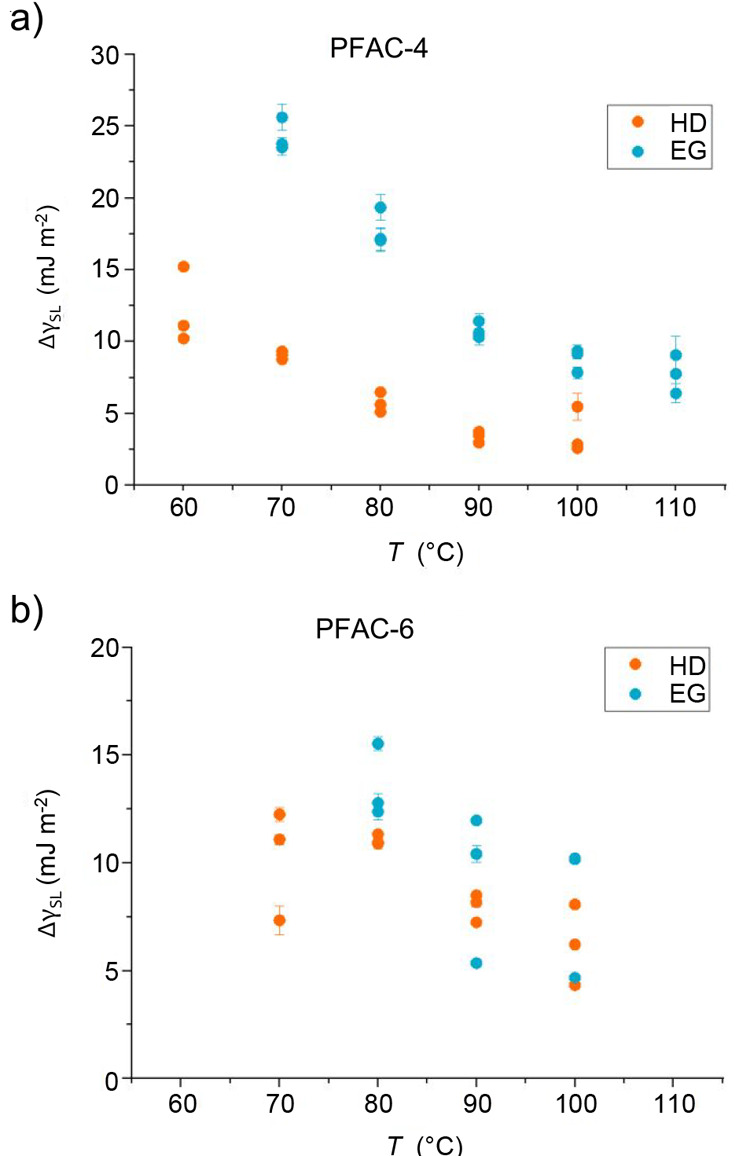
Calculated change in solid–liquid surface energy, Δγ_SL_, induced by EG (blue) and HD (orange) for (a) PFAC-4 and (b) PFAC-6. Δγ_SL_ is defined as the difference between values at *t* = 0 and *t* = ∞. Note the different axes

Finally, the values of Δγ_SL_ were calculated, defined as the difference between γ_SL_(0) and γ_SL_(∞), and these are plotted in Figure 10. The results for each measurement is plotted separately with the error bars indicating goodness of fit. For PFAC-4, there is a definite difference between EG and HD, with the values for HD being lower at any given value of *T*. This is as expected since EG engages in strong polar interactions with the surface in comparison with the purely dispersive HD. There is also a very evident decrease in Δγ_SL_ with increasing *T*. This is because the configuration of the surface at *t* = 0 transitions from the side chains being oriented with CF_3_ at the surface (low-*T* regime) to isotropy (high-*T* regime). The increased presence of acrylate groups at the surface in the isotropic configuration is closer in energy to the configuration at *t* = ∞ where all of the acrylate groups are at the surface with CF_3_ groups buried. The results for PFAC-6 are less clear-cut, having a larger scatter and no obvious differentiation between the two liquids. There does appear to be a general decrease in the values of Δγ_SL_ with *T*, but it is not pronounced.

It is difficult to make a valid comparison between PFAC-4 and -6 since the values of γ_SL_(0) and γ_SL_(∞) behave differently as a function of *T*; the surface energy of an isotropic surface is dependent on the stoichiometry of the different functional groups in the polymer and the temperature at which a surface achieves isotropy is dependent on the strength of interactions between those functional groups. Having a longer fluorinated side chain, we would therefore expect isotropic PFAC-6 to have a higher value of γ_SL_ (in this experiment) and a lower γ_SV_. Stronger interactions between the side chains give a higher value of *T* at which the surface achieves isotropy in comparison to PFAC-4.

## Conclusions

The contact angles of ethylene glycol and hexadecane on PFAC-*n* polymer melts display time dependency, which we conclude to be the result of surface reconstruction. The driving force for this reorganization is the polar interactions between ethylene glycol and the ester group in the polymer, and the stronger dispersive interactions between hexadecane and the hydrocarbon groups in the polymer. The latter effect is of particular interest and has not been reported elsewhere.

The first order kinetics model proposed by Butt et al. is able to predict the contact angle aging on PFAC-4 and -6 provided that the relaxation time falls approximately within the experimental time frame. Outside of this time frame, it is assumed that the model continues to be valid but cannot conclusively be stated. Likewise, the relaxation times for PFAC-8 above its melting point of 70 °C longer than the experimental time frame and cannot be modeled. The relaxation times in the region where the model is valid are of the order of seconds, which is consistent with a reorientation process at the solid surface.

Our investigation has focused on the commonly used PFAC-*n* system; nonetheless, there are wide ranging implications in terms of surface design, in terms of designing both oleo/fluorophobic surfaces or smart surfaces which rearrange in response to hydrocarbons or fluorocarbons. The deeper understanding of surface properties of these PFAC-*n* surfaces will contribute toward the development of new oleophilic and smart coatings beyond the most commonly used PFAC-10, as this is phased out of use for environmental reasons, including development of nonfluorinated coatings.

## Supporting Information Available

Additional characterization of plasma polymerized PFAC-n surface (PDF)
Complete contact angle aging data and data fitted using Butt’s first order model (PDF)

## Abbreviations

AFM: atomic force microscopy
CA: contact angle
DSC: differential scanning calorimetry
EG: ethylene glycol
HD: *n-*hexadecane
PFAC-*n*: perfluoro-*n-*yl acrylate
SE: surface energy
XPS: X-ray photoelectron spectroscopy
