# Supplementary material for: Surface Reconstruction of Fluoropolymers in Liquid Media
Source: Langmuir. 2022 Apr 8;38(15):4657–68. doi: 10.1021/acs.langmuir.2c00198 (PMC9097541; doi:10.1021/acs.langmuir.2c00198)
Supplement: Supplementary file 1 — la2c00198_si_001.pdf [file la2c00198_si_001.pdf]

The surface reconstruction of fluoropolymers in liquid media

Supporting Information 1: Detailed characterization of plasma polymerized PFAC-n surfaces

Eleanor Milnes-Smith,[a] Corinne A. Stone,[b] Colin R. Willis,[b] and Susan Perkin[a]*

[a] *Department of Chemistry, Physical and Theoretical Chemistry Laboratory, University of Oxford, Oxford OX1 3QZ, UK*

[b] *Defence Science and Technology Laboratory, Porton Down, Salisbury, Wiltshire SP4 0JQ, UK*

* Corresponding Author

Email: susan.perkin@chem.ox.ac.uk

Contents

Fig S1.1 Solid-state ^{19}F NMR spectra of PFAC-*n* polymers
Fig S1.2 Deconvoluted C(1s) XPS spectra of PFAC-*n* polymers
Fig S1.3 Angle-resolved XPS C(1s) spectra of PFAC-*n* polymer surfaces
Fig S1.4 Variation with depth of the concentration of different carbon species in PFAC-n surfaces
Fig S1.5 Variation with depth of the concentration of CF_3 and C—H carbon species in PFAC-n surfaces
Fig S1.6 DSC traces of PFAC-8 and -10
Fig S1.7 AFM micrographs of PFAC-8 and PFAC-10

Table S1.1 Peak positions and assignments from the deconvoluted C(1s) XPS spectra

Solid-state ^{19}F NMR results

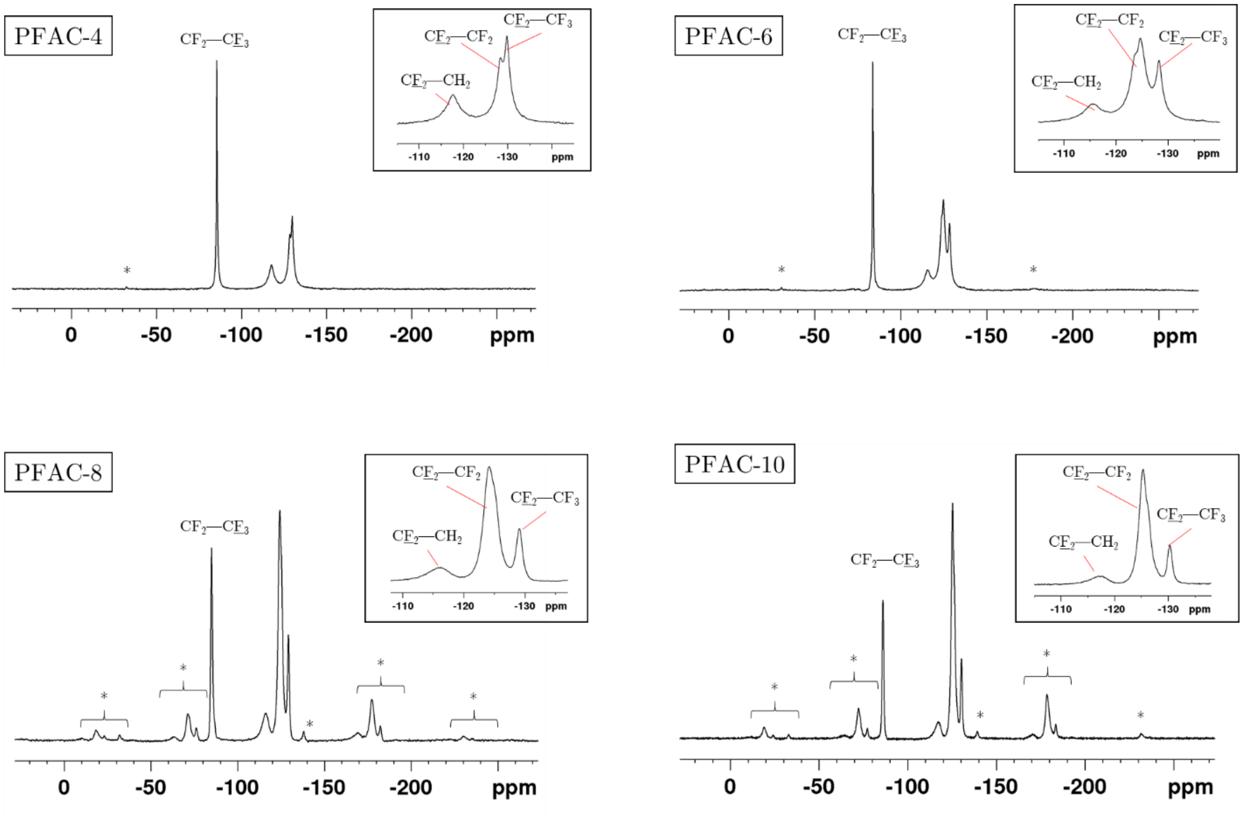

Figure S1.1 Solid-state ^{19}F NMR spectra of PFAC-*n* surfaces acquired with a magic angle spinning frequency of 20 kHz. Asterisks indicate sidebands

Carbon 1s XPS results

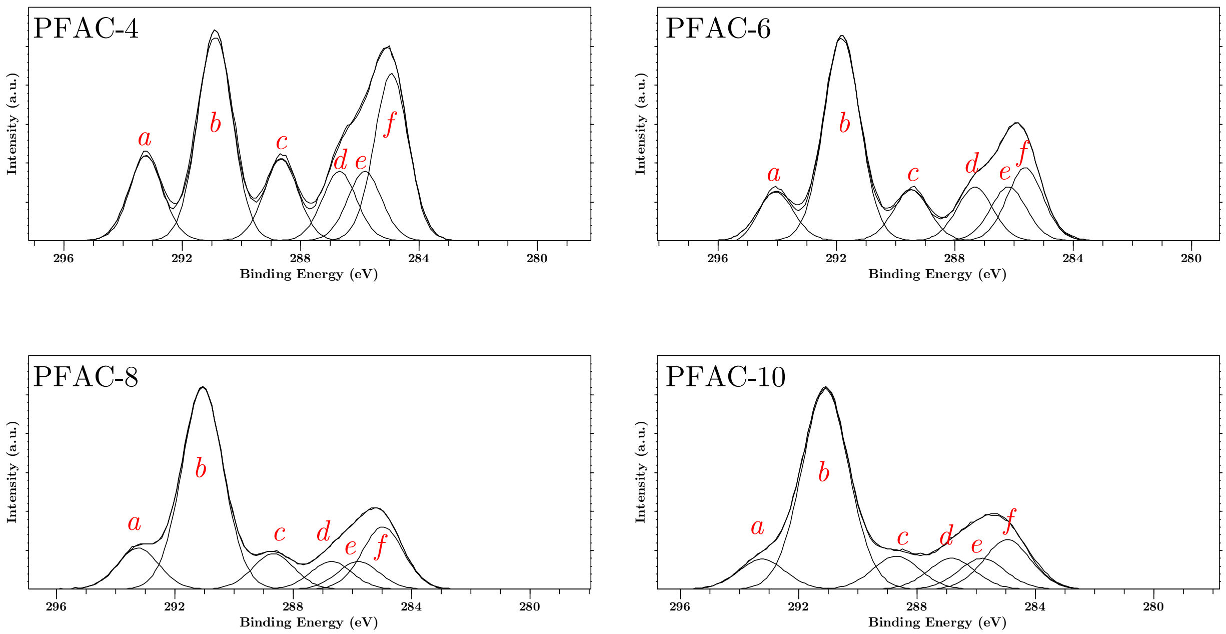

Figure S1.2 Deconvoluted C(1s) XPS spectra of PFAC-*n* polymers acquired at a take-off angle normal to the surface and using a Shirley background subtraction. The letters correspond to the peak assignments in Table S1.1.

**PFAC-*n***	**Peak Position (eV)**					
	**CF_3 *(a)***	**CF_2 *(b)***	**C=O *(c)***	**C—O *(d)***	**<u>C</u>—CF_n *(e)***	**<u>C</u>—CH_n *(f)***
4	293.3	291.0	288.7	286.8	285.9	285.0
6	293.4	291.2	288.9	286.8	285.8	285.0
8	293.3	291.1	288.7	286.7	285.8	285.0
10	293.2	291.1	288.7	286.8	285.8	285.0

Table S1.1 Peak positions and assignments from the deconvoluted C(1s) XPS spectra. The hydrocarbon peak position is calibrated to be 285.0 eV. The FWHM are PFAC-4 (1.35 eV), PFAC-6 (1.44 eV), PFAC-8 (1.64 eV), and PFAC-10 (1.86 eV). The letters in superscript correspond to the marked peaks in Figure S1.2

Angle-Resolved Carbon 1s XPS results

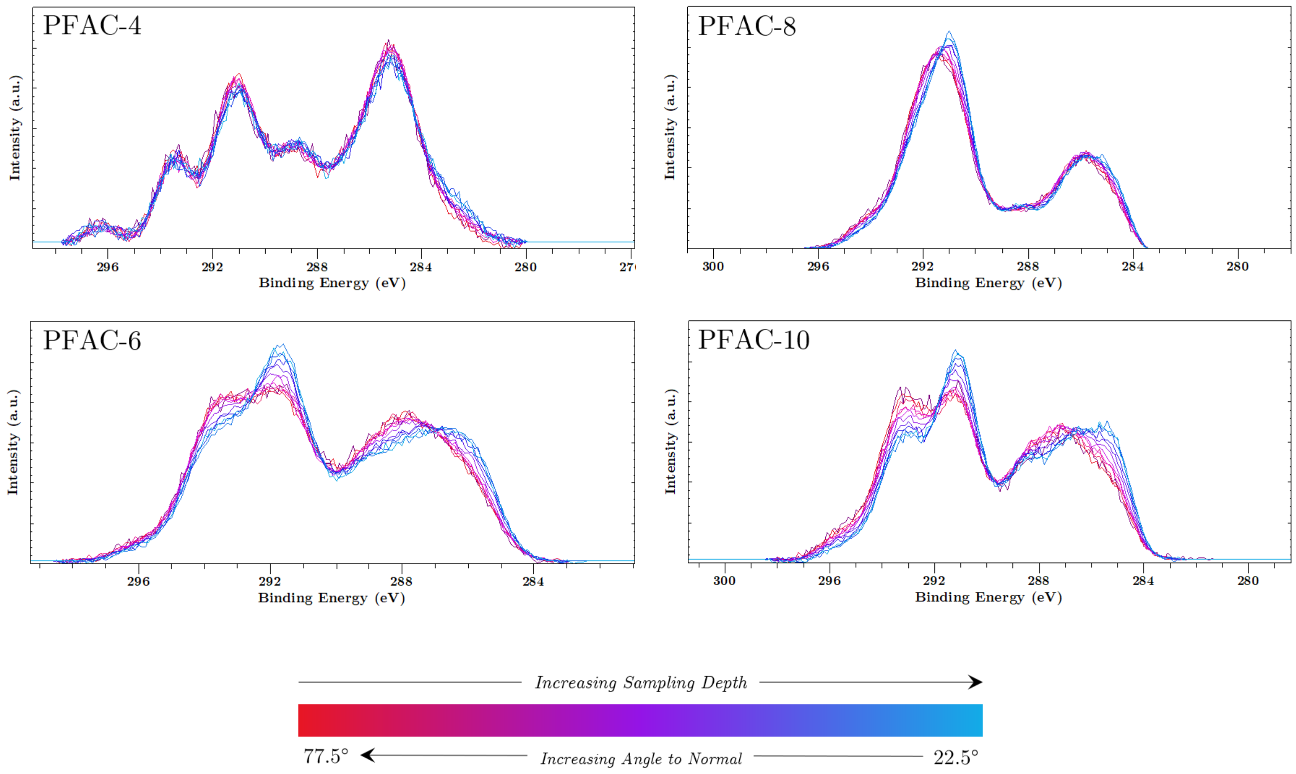

Figure S1.3 Angle-resolved XPS carbon 1s spectra of PFAC-*n* polymer surfaces acquired in parallel at angles between 77.5° (red) and 22.5° (blue) from the surface normal in intervals of 5°. To account for the decrease in in signal intensity with increasing angle to the surface normal, a Shirley background subtraction has been performed, and the area under each of the peaks have been normalized. In all cases, there is an overabundance of the low energy CF_3 group near the surface, and an underabundance of the higher energy hydrocarbon group. This effect is more apparent by eye for PFAC-6 and PFAC-10 than it is for the other two polymers. To assist with deconvolution, the normalized spectra were summed across all angles, and peak fitting carried out on the resulting spectrum with each peak being constrained to have equal full-width half-maxima (FWHM). The peak positions were then fixed and exported to the individual spectra where the FWHM and relative peak intensity was adjusted to get the best possible fit

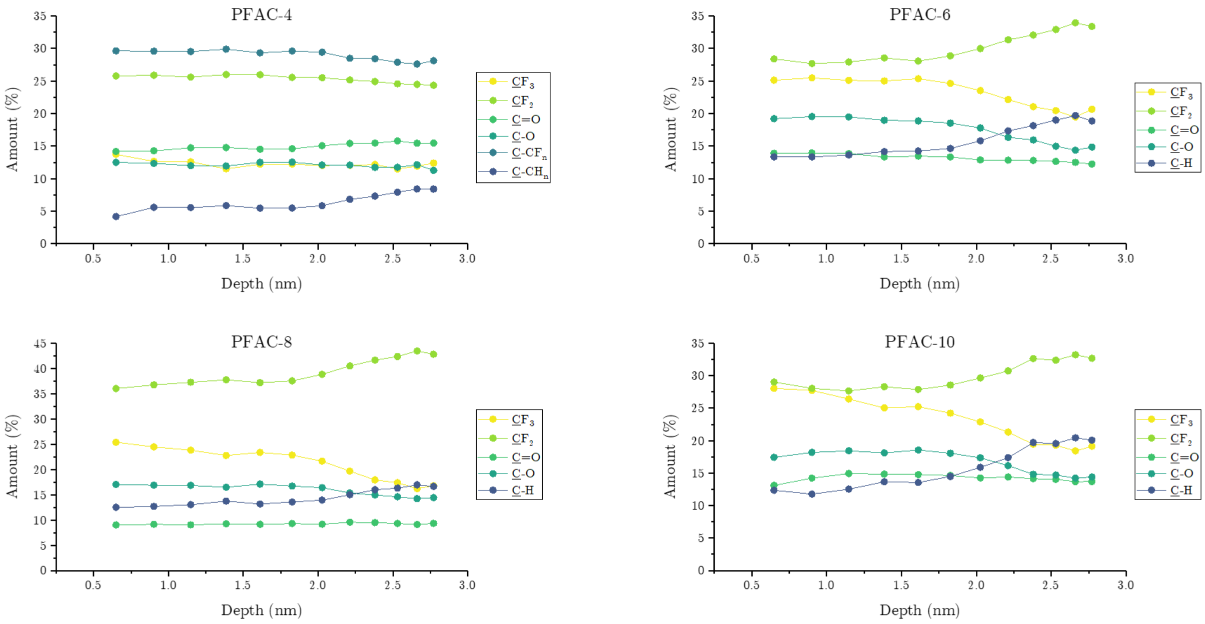

Figure S1.4 Variation with penetration depth, d, of the concentration of different carbon species in PFAC-n surfaces as determined via ARXPS (note the different y axes). The penetration depth is defined as the depth at which 63% of the signal originates and is estimated as $d \sim \sin\theta$ where θ is the take-off angle relative to the surface, and λ is the electron inelastic mean free path. For fluoropolymers where the photoelectrons are excited from the C 1s level by Al Kα radiation, λ ~ 3nm. Lines are added to guide the eye.

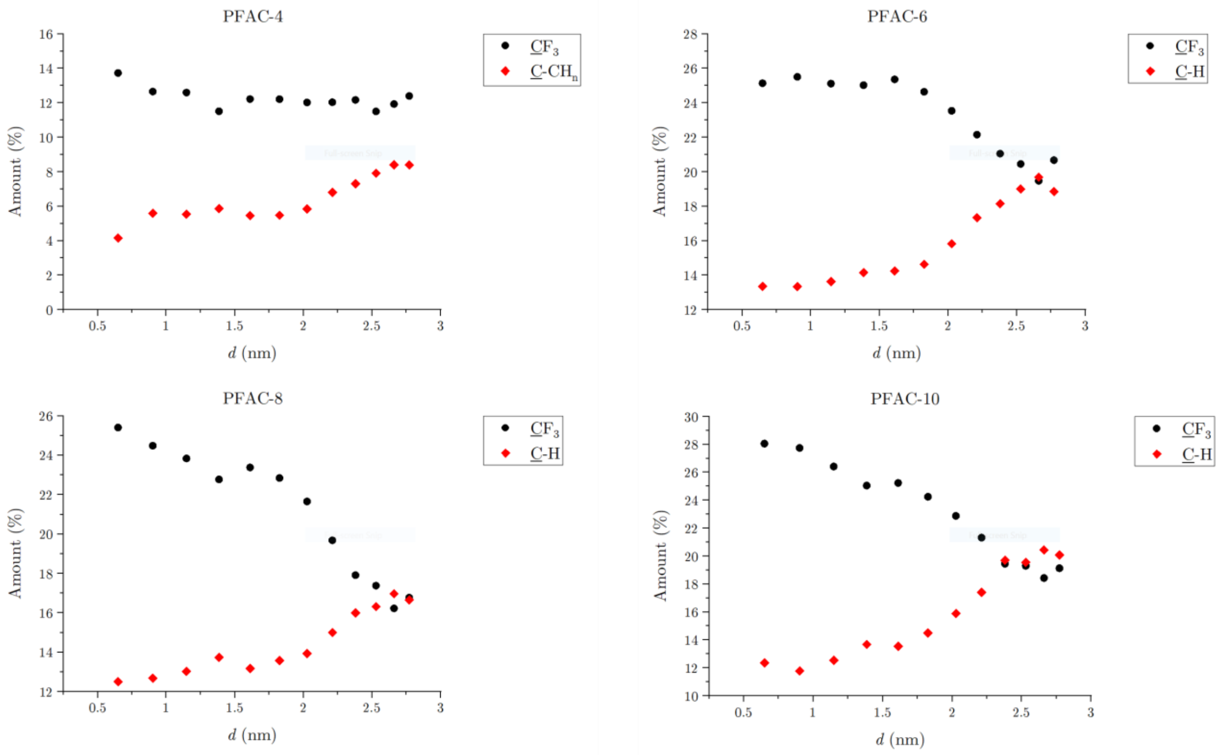

Figure S1.5 Variation with penetration depth, d, of the concentration of CF3 and C—H carbon species in PFAC-n surfaces as determined via ARXPS . This data is the same as that in Figure S1.4

Differential Scanning Calorimetry (DSC)

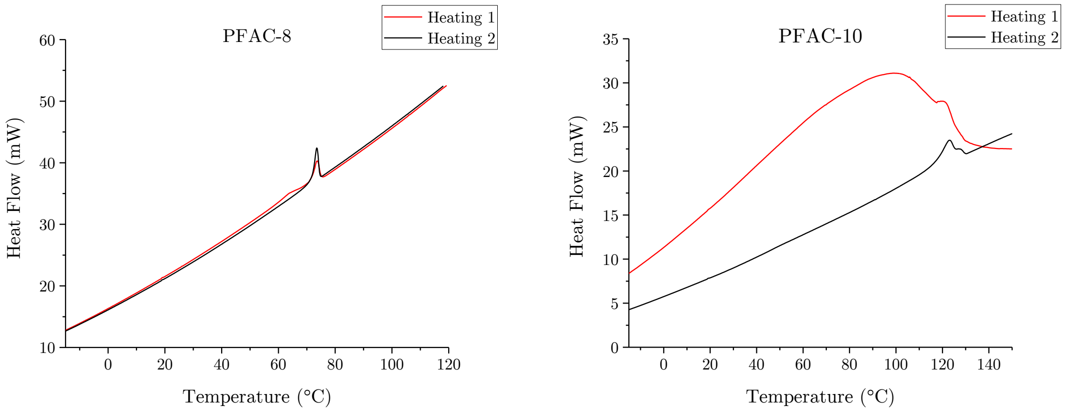

Figure S1.6 DSC traces of PFAC-8 and -10 showing heat flow with temperature for the first (red) and second (black) heating cycles. These traces are shown endothermic up

Atomic Force Microscopy (AFM)

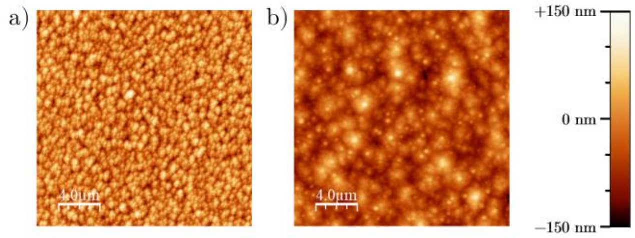

Figure S1.7 20 x 20 μm^2 AFM micrographs of a) PFAC-8 and b) PFAC-10. The images have been subjected to a 2nd order flattening to remove bow from the scanner motion
