# Supplementary material for: Surface Reconstruction of Fluoropolymers in Liquid Media
Source: Langmuir. 2022 Apr 8;38(15):4657–68. doi: 10.1021/acs.langmuir.2c00198 (PMC9097541; doi:10.1021/acs.langmuir.2c00198)
Supplement: Supplementary file 2 — la2c00198_si_002.pdf [file la2c00198_si_002.pdf]

The surface reconstruction of fluoropolymers in liquid media

Supporting Information 2: Complete contact angle aging data and data fitted using Butt's first order model

Eleanor Milnes-Smith,[a] Corinne A. Stone,[b] Colin R. Willis,[b] and Susan Perkin[a]*

[a] *Department of Chemistry, Physical and Theoretical Chemistry Laboratory, University of Oxford, Oxford OX1 3QZ, UK*

[b] *Defence Science and Technology Laboratory, Porton Down, Salisbury, Wiltshire SP4 0JQ, UK*

* Corresponding Author

Email: susan.perkin@chem.ox.ac.uk

Contents

Fig S2.1 Variation of contact angles with time of drops of ethylene glycol and hexadecane on PFAC-n- surfaces
Fig S2.2 a) – e) Contact aging data for ethylene glycol on PFAC-4 fitted to Butt's first order model
Fig S2.3 a) – e) Contact aging data for *n*-hexadecane on PFAC-4 fitted to Butt's first order model
Fig S2.4 a) – c) Contact aging data for ethylene glycol on PFAC-6 fitted to Butt's first order model
Fig S2.5 a) – d) Contact aging data for *n*-hexadecane on PFAC-6 fitted to Butt's first order model

Contact Angle Aging Results

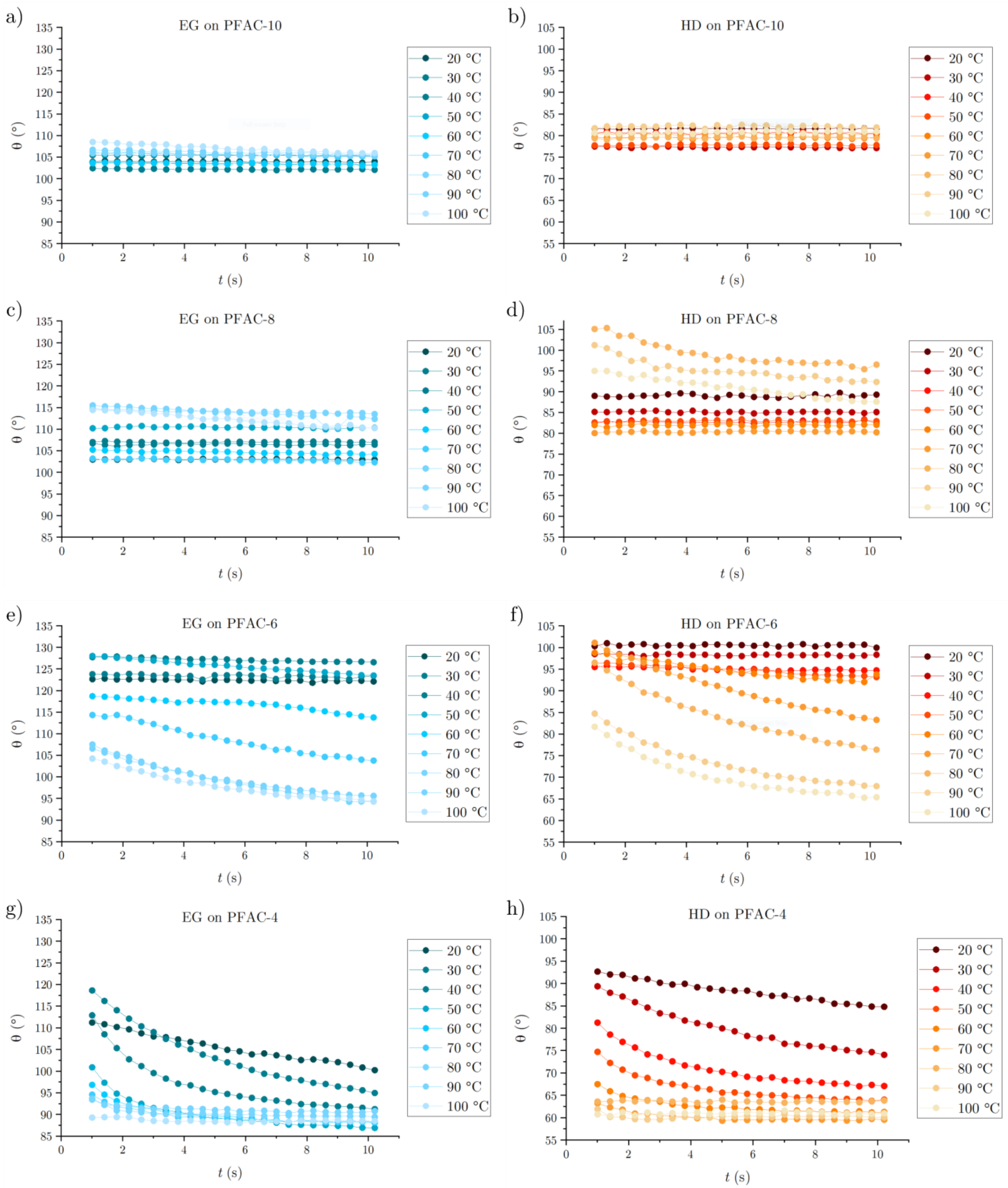

Figure S2.1 Variation of the static contact angles, θ, with time, t, of a single representative drops of ethylene glycol (EG, blue) and hexadecane (HD, orange) on PFAC-n- surfaces at $20 \leq T \leq 100$.

Data Fitted Using Butt's First Order Model

All data was fitted using Eq. 3 - $\cos\theta(t) = \cos\theta(\infty) + \Delta\cos\theta\, e^{-t/\tau_{SL}}$

Ethylene Glycol on PFAC-4

S2.2 a)

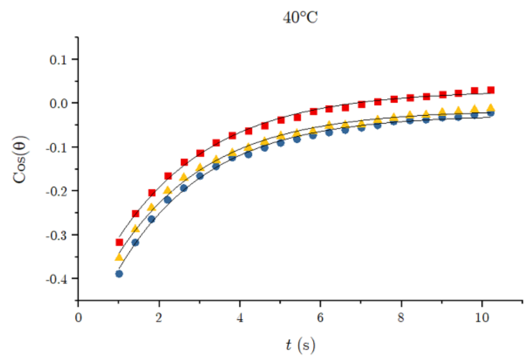

Parameter	Value	Standard Error
$\cos\theta(\infty)$	0.0296	0.0030
$\Delta\cos\theta$	-0.5090	0.0101
τ	2.3963	0.0817
$\cos\theta(\infty)$	-0.0266	0.0030
$\Delta\cos\theta$	-0.5489	0.0120
τ	2.2378	0.0772
$\cos\theta(\infty)$	-0.0151	0.0031
$\Delta\cos\theta$	-0.5042	0.0114
τ	2.3093	0.0851

S2.2 b)

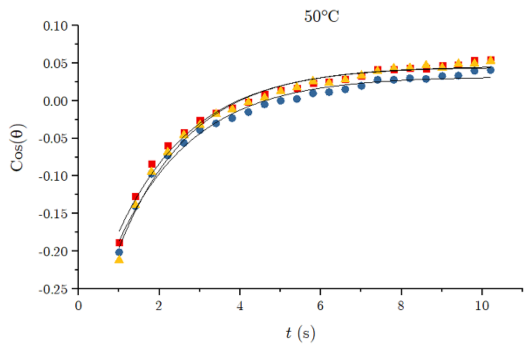

Parameter	Value	Standard Error
$\cos\theta(\infty)$	0.0462	0.0030
$\Delta\cos\theta$	-0.3746	0.0175
τ	1.8858	0.1154
$\cos\theta(\infty)$	0.0323	0.0030
$\Delta\cos\theta$	-0.3726	0.0175
τ	1.8881	0.1163
$\cos\theta(\infty)$	0.0449	0.0030
$\Delta\cos\theta$	-0.4226	0.0199
τ	1.7797	0.1034

S2.2 c)

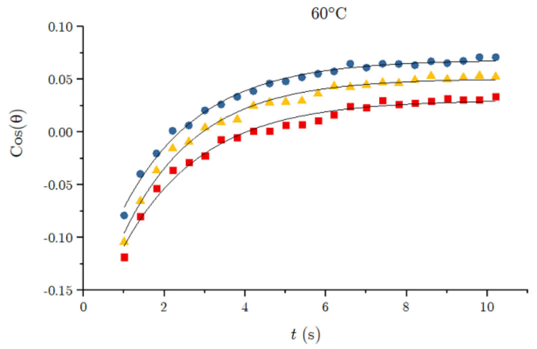

Parameter	Value	Standard Error
$\cos\theta(\infty)$	0.0303	0.0023
$\Delta\cos\theta$	-0.2297	0.0119
τ	1.9861	0.1414
$\cos\theta(\infty)$	0.0685	0.0014
$\Delta\cos\theta$	-0.2363	0.0080
τ	1.9230	0.0866
$\cos\theta(\infty)$	0.0506	0.0018
$\Delta\cos\theta$	-0.2546	0.0114
τ	1.8267	0.1038

S2.2 d)

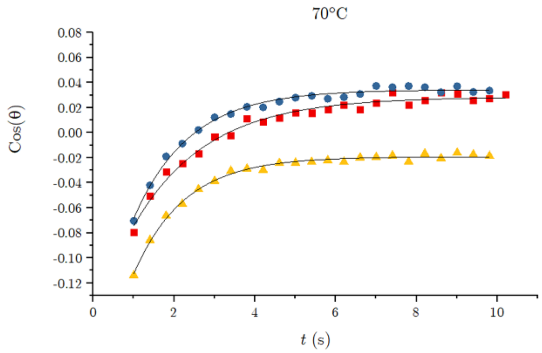

Parameter	Value	Standard Error
$\cos\theta(\infty)$	0.0284	0.0014
$\Delta\cos\theta$	-0.1786	0.0092
τ	1.8218	0.1184
$\cos\theta(\infty)$	0.0342	0.0009
$\Delta\cos\theta$	-0.2126	0.0098
τ	1.3814	0.0636
$\cos\theta(\infty)$	-0.0192	0.0006
$\Delta\cos\theta$	-0.2084	0.0085
τ	1.2547	0.0472

S2.2 e)

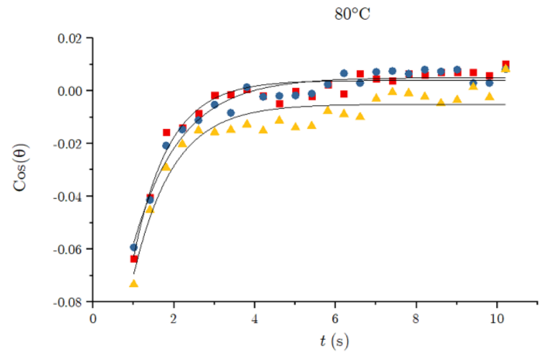

Parameter	Value	Standard Error
$\cos\theta(\infty)$	0.00394	9.63E-04
$\Delta\cos\theta$	-0.21064	0.03066
τ	0.87705	0.08958
$\cos\theta(\infty)$	0.00502	9.30E-04
$\Delta\cos\theta$	-0.14846	0.01547
τ	1.16922	0.10599
$\cos\theta(\infty)$	-0.0051	0.00145
$\Delta\cos\theta$	-0.1805	0.03571
τ	0.97885	0.14749

***n*-Hexadecane on PFAC-4**

S2.3 a)

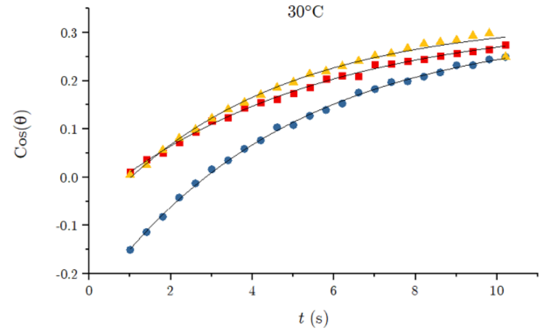

Parameter	Value	Standard Error
$\cos\theta(\infty)$	0.4258	0.0026
$\Delta\cos\theta$	-0.3910	0.0049
τ	3.0287	0.0898
$\cos\theta(\infty)$	0.4158	0.0024
$\Delta\cos\theta$	-0.2101	0.0074
τ	2.5111	0.1603
$\cos\theta(\infty)$	0.4255	0.0012
$\Delta\cos\theta$	-0.3009	0.0029
τ	2.7897	0.0557

S2.3 b)

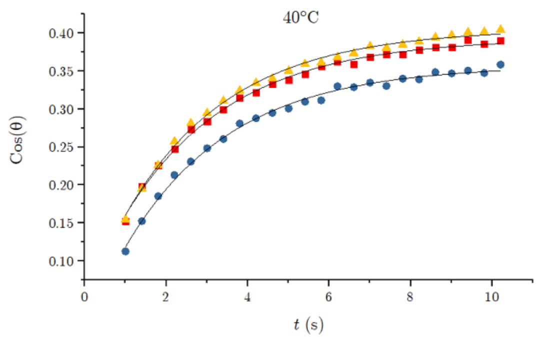

Parameter	Value	Standard Error
$\cos\theta(\infty)$	0.3572	0.0025
$\Delta\cos\theta$	-0.3515	0.0070
τ	2.6008	0.0988
$\cos\theta(\infty)$	0.3932	0.0023
$\Delta\cos\theta$	-0.3402	0.0061
τ	2.6408	0.0923
$\cos\theta(\infty)$	0.4055	0.0023
$\Delta\cos\theta$	-0.3618	0.0066
τ	2.5855	0.0888

S2.3 c)

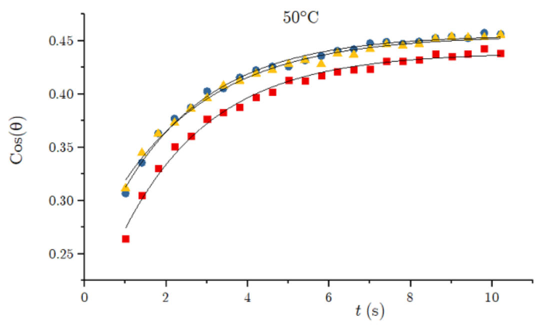

Parameter	Value	Standard Error
$\cos\theta(\infty)$	0.4388	0.0021
$\Delta\cos\theta$	-0.2604	0.0086
τ	2.2050	0.1133
$\cos\theta(\infty)$	0.4554	0.0016
$\Delta\cos\theta$	-0.2259	0.0068
τ	2.2022	0.1027
$\cos\theta(\infty)$	0.4550	0.0019
$\Delta\cos\theta$	-0.2057	0.0062
τ	2.4273	0.1264

S2.3 d)

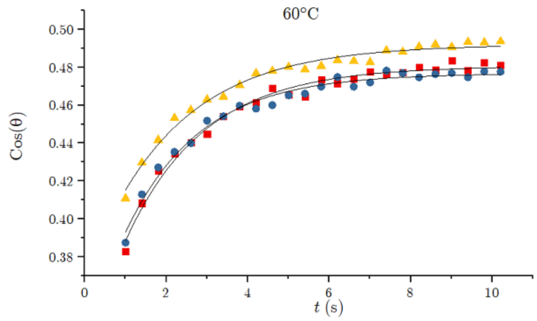

Parameter	Value	Standard Error
$\cos\theta(\infty)$	0.4920	0.0012
$\Delta\cos\theta$	-0.1232	0.0054
τ	2.1237	0.1385
$\cos\theta(\infty)$	0.4806	0.0013
$\Delta\cos\theta$	-0.1551	0.0070
τ	1.9355	0.1170
$\cos\theta(\infty)$	0.4767	0.0012
$\Delta\cos\theta$	-0.1456	0.0073
τ	1.8247	0.1166

S2.3 e)

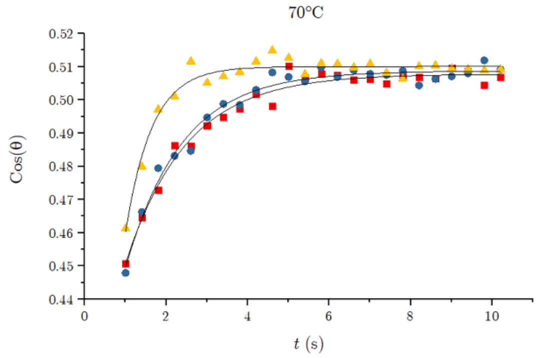

Parameter	Value	Standard Error
$\cos\theta(\infty)$	0.5100	0.0006
$\Delta\cos\theta$	-0.2358	0.0404
τ	0.6486	0.0619
$\cos\theta(\infty)$	0.5078	0.0008
$\Delta\cos\theta$	-0.1118	0.0078
τ	1.4965	0.1107
$\cos\theta(\infty)$	0.5087	0.0007
$\Delta\cos\theta$	-0.1226	0.0087
τ	1.3818	0.0972

Ethylene Glycol on PFAC-6

S2.4 a)

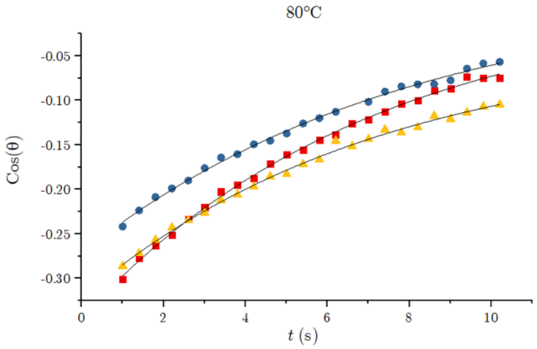

Parameter	Value	Standard Error
$\cos\theta(\infty)$	0.0158	0.0096
$\Delta\cos\theta$	-0.3605	0.0074
τ	7.1373	0.3926
$\cos\theta(\infty)$	0.0249	0.0129
$\Delta\cos\theta$	-0.2966	0.0104
τ	8.0413	0.6707
$\cos\theta(\infty)$	-0.0348	0.0118
$\Delta\cos\theta$	-0.2873	0.0091
τ	7.2269	0.6080

S2.4 b)

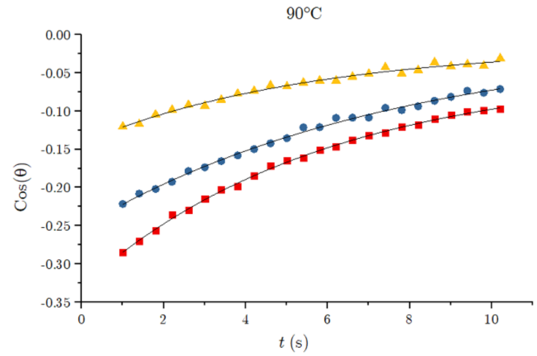

Parameter	Value	Standard Error
$\cos\theta(\infty)$	-0.0459	0.0048
$\Delta\cos\theta$	-0.2836	0.0034
τ	5.8795	0.2372
$\cos\theta(\infty)$	-0.0055	0.0114
$\Delta\cos\theta$	-0.2467	0.0091
τ	7.6760	0.7002
$\cos\theta(\infty)$	-0.0149	0.0064
$\Delta\cos\theta$	-0.1269	0.0044
τ	5.5799	0.6955

S2.4 c)

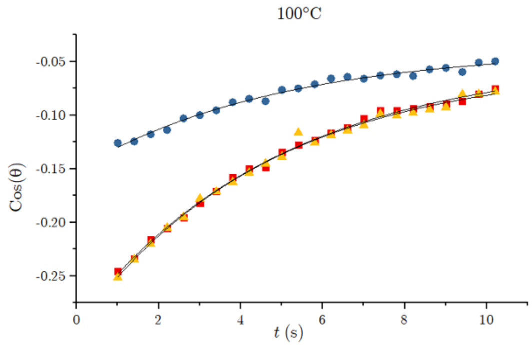

Parameter	Value	Standard Error
$\cos\theta(\infty)$	-0.0464	0.0042
$\Delta\cos\theta$	-0.2473	0.0029
τ	4.9368	0.2278
$\cos\theta(\infty)$	-0.0381	0.0044
$\Delta\cos\theta$	-0.1131	0.0030
τ	4.9086	0.5197
$\cos\theta(\infty)$	-0.0533	0.0055
$\Delta\cos\theta$	-0.2461	0.0040
τ	4.6248	0.2980

n-Hexadecane on PFAC-6

S2.5 a)

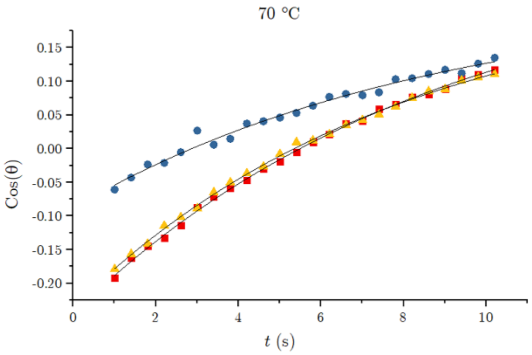

Parameter	Value	Standard Error
$\cos\theta(\infty)$	0.2282	0.0341
$\Delta\cos\theta$	-0.3164	0.0286
τ	8.7892	1.7226
$\cos\theta(\infty)$	0.2824	0.0168
$\Delta\cos\theta$	-0.5280	0.0140
τ	8.7798	0.5072
$\cos\theta(\infty)$	0.2422	0.0131
$\Delta\cos\theta$	-0.4779	0.0105
τ	7.8732	0.4172

S2.5 b)

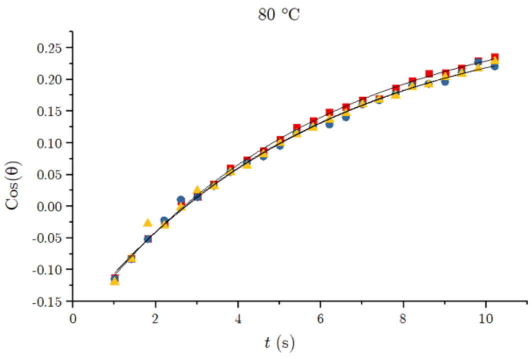

Parameter	Value	Standard Error
$\cos\theta(\infty)$	0.3149	0.0084
$\Delta\cos\theta$	-0.5071	0.0058
τ	5.6309	0.2295
$\cos\theta(\infty)$	0.3074	0.0140
$\Delta\cos\theta$	-0.4904	0.0098
τ	5.8613	0.3984
$\cos\theta(\infty)$	0.3045	0.0153
$\Delta\cos\theta$	-0.4875	0.0105
τ	5.7686	0.4343

S2.5 c)

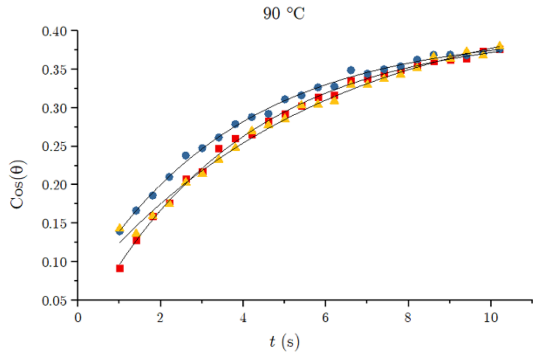

Parameter	Value	Standard Error
$\cos\theta(\infty)$	0.3994	0.0039
$\Delta\cos\theta$	-0.3956	0.0041
τ	3.7581	0.1306
$\cos\theta(\infty)$	0.3976	0.0038
$\Delta\cos\theta$	-0.3373	0.0041
τ	3.7307	0.1499
$\cos\theta(\infty)$	0.4443	0.0143
$\Delta\cos\theta$	-0.3805	0.0099
τ	5.7590	0.5201

S2.5 d)

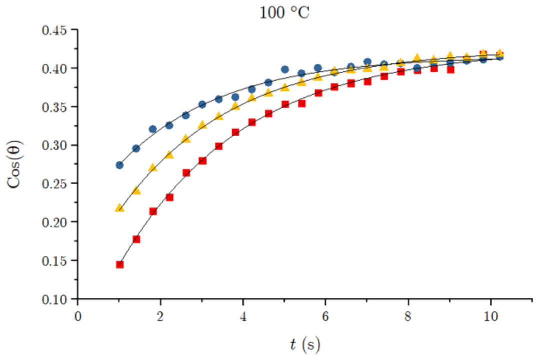

Parameter	Value	Standard Error
$\cos\theta(\infty)$	0.4258	0.0026
$\Delta\cos\theta$	-0.3910	0.0049
τ	3.0287	0.0898
$\cos\theta(\infty)$	0.4158	0.0024
$\Delta\cos\theta$	-0.2101	0.0074
τ	2.5111	0.1603
$\cos\theta(\infty)$	0.4255	0.0012
$\Delta\cos\theta$	-0.3009	0.0029
τ	2.7897	0.0557
